# Molecular Bases of Fruit Quality in *Prunus* Species: An Integrated Genomic, Transcriptomic, and Metabolic Review with a Breeding Perspective

**DOI:** 10.3390/ijms22010333

**Published:** 2020-12-30

**Authors:** Beatriz E. García-Gómez, Juan A. Salazar, María Nicolás-Almansa, Mitra Razi, Manuel Rubio, David Ruiz, Pedro Martínez-Gómez

**Affiliations:** 1 Department of Plant Breeding, CEBAS-CSIC, P.O. Box 164, 30100 Murcia, Spain; begomez@cebas.csic.es (B.E.G.-G.); jasalazar@cebas.csic.es (J.A.S.); mnicolas@cebas.csic.es (M.N.-A.); mrubio@cebas.csic.es (M.R.); druiz@cebas.csic.es (D.R.); 2 Department of Horticulture, Faculty of Agriculture, University of Zajan, Zanjan 45371-38791, Iran; razi.mitra@alumni.znu.ac.ir

**Keywords:** *Prunus*, fruit quality, genomics, transcriptomics, metabolomics, epigenetics, breeding

## Abstract

In plants, fruit ripening is a coordinated developmental process that requires the change in expression of hundreds to thousands of genes to modify many biochemical and physiological signal cascades such as carbohydrate and organic acid metabolism, cell wall restructuring, ethylene production, stress response, and organoleptic compound formation. In *Prunus* species (including peaches, apricots, plums, and cherries), fruit ripening leads to the breakdown of complex carbohydrates into sugars, fruit firmness reductions (softening by cell wall degradation and cuticle properties alteration), color changes (loss of green color by chlorophylls degradation and increase in non-photosynthetic pigments like anthocyanins and carotenoids), acidity decreases, and aroma increases (the production and release of organic volatile compounds). Actually, the level of information of molecular events at the transcriptional, biochemical, hormonal, and metabolite levels underlying ripening in *Prunus* fruits has increased considerably. However, we still poorly understand the molecular switch that occurs during the transition from unripe to ripe fruits. The objective of this review was to analyze of the molecular bases of fruit quality in *Prunus* species through an integrated metabolic, genomic, transcriptomic, and epigenetic approach to better understand the molecular switch involved in the ripening process with important consequences from a breeding point of view.

## 1. Introduction

In the plant kingdom, fruit ripening is a coordinated process that requires the change in expression of hundreds to thousands of genes to modify many biochemical and physiological signals including carbohydrate and organic acids metabolism, cell wall restructuring, ethylene production, stress response, and organoleptic compound formation. Several studies have tried to comprehend the main processes that take place during the transformation between a green fruit to ripe fruit, understanding horticultural maturity as the moment when the “plant or plant part possesses the prerequisites for utilization by consumers for a particular purpose” [1]. Ripening was also described by this author as “the composite of processes that occur from the later stages of growth and development through the early stages of senescence and that results in characteristic aesthetic and/or food quality, as evidenced by changes in composition, color, texture, or other sensory attributes” [1]. Ripening is a well-known step of fruit development and has been extensively studied because of the importance of accurately describing the exact point where the fruit expresses greater nutritional and economic value. Senescence is the process that follows fruit ripening and leads to the death of tissue. Though it is not lethal, senescence increases the susceptibility to disease, injury, dehydration, or microbial invasion. It is hard to discriminate between these stages since they sometimes overlap, but ripening includes processes like pigment accumulation and cell wall changes that are not usually involved in senescence processes [2].

**Figure 1:**
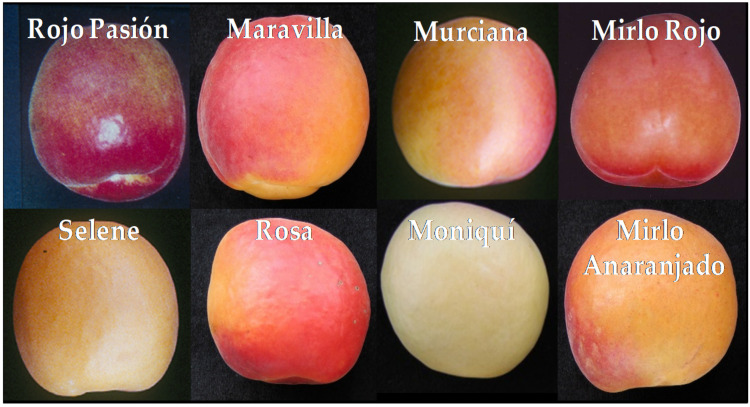
Traditional and new Spanish apricot cultivars at consumer ripening state.

Fruit ripening leads to the breakdown of complex carbohydrates into sugars, fruit firmness reductions (softening by cell wall degradation and cuticle properties alteration), color changes (loss of green color by chlorophylls degradation and increase in non-photosynthetic pigments like anthocyanins and carotenoids), acidity decreases, and aroma increases (production and release of organic volatile compounds) [3,4]. This process contributes to increased attractiveness and acceptance by consumers (Figure 1). Mainly, the composition of sugars, acids, color, and aroma depends on genetics; however, culture practices and environmental factors strongly influence fruit quality characters [5]. Understanding ripening mechanisms will make it feasible to implement agronomical strategies optimized to climatic conditions, enhance production, increase fruit quality, and facilitate the selection of new apricot varieties. Therefore, in this review, ripening was extensively studied because of the importance of defining exactly when a fruit displays the highest nutritional and economic value, thus rendering fruits more attractive and palatable due to the acquisition of fruit quality traits.

Additionally, fruit development is a regulated process unique to plants that involves three distinct steps: fruit set, fruit growth, and ripening. Fruit development finishes when the fruit is ripe and is defined as the optimum moment for harvest the fruit from the point of view of the consumer. Each step is controlled by genetic and environmental factors, influenced by culture practices. Each of these factors interacts in a multilevel process and triggers the coordinated action of master regulators, including hormone signaling, microRNAs, and epigenetic modifying genes [6]. On the other hand, the epigenetic regulation of gene expression has been recognized as remodeling gene expression during the ripening process [7,8], mainly by the DNA methylation of targeted loci involved in gene expression regulation. The presence of tissue-specific methylation patterns and the progressive demethylation of ripening-related gene promoters give tissue specificity and developmental stage-dependent gene expression that may be used as a strategy to monitoring ripening process [8,9].

**Figure 2:**
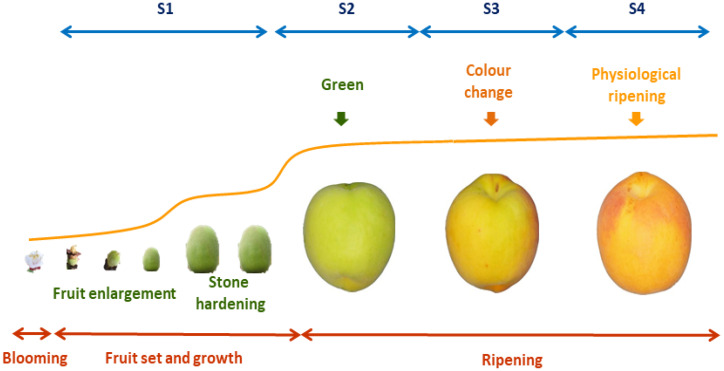
Stages of fruit development and ripening in apricot fruits. After flower bloom, pollination and fruit set, fruit growth begin with a fruit enlargement, stopping the size increase during stone hardening (S1). The reactivation of grown at green stage (S2) is followed by a color change in half-ripe fruit (S3). Maturation and ripening end at physiological ripening, when the fruit reaches the maximum sucrose accumulation and definitive color (S4).

In *Prunus* species including peaches, nectarines, (*Prunus persica* (L.) Batsch), prunes (*Prunus domestica* L.), Japanese plums (*Prunus salicina* Lindl), apricots (*Prunus armeniaca* L.) and sweet (*Prunus avium* L.) (2n = 2x = 16) and sour (*Prunus cerasus* L.) cherry fruits, four stages have been described during fruit development and ripening: S1: fruit growth; S2: green fruit; S3: changing color; and S4: physiological ripening (Figure 2). The beginning of S1 is characterized by fruit set and growth. A decrease in fruit growth at the S1/S2 transition is followed by endocarp lignification (stone hardening), from the middle of S2 to its end. The S3 phase begins with additional growth activation, mainly due to the increase in the number of cells, thus generating the second exponential growth phase. The maturation of the fruit is completed at the end of S3, and then the last ripening phase S4 occurs when the fruit fully develops its quality traits. The fruit developmental phases are determined using a mathematical model based on the first derivative of the growth cumulative curve, represented by a double sigmoid pattern. The identification and characterization of each phase of fruit growth are necessary for development studies and the precision harvesting of high quality fruits [10].

On the other hand, fruits have been classified into two physiological groups with two distinct ripening fruit mechanisms—climacteric and non-climacteric—based on the presence or absence of a ripening-associated rise of ethylene and an increase of the respiration ratio. In climacteric fruits, such as *Prunus* species, the ripening process is marked by an increased respiration ratio induced by ethylene, while in non-climacteric fruits, it is controlled by an ethylene-independent process with little change in the respiration rate [11]. The attribute acquisition during the ripening process in climacteric fruit differs among species, varieties, nutritional feeds, and environments. The most significant changes during ripening are represented in Table 1 [2,3]. In the final phase of fruit ripening, oxidative processes with the accumulation of hydrogen peroxide and membrane lipid peroxidation occur. The identification of enzymes from the cell antioxidant systems including catalase (CAT), peroxidase (POX), and superoxide dismutase (SOD)involved in the scavenging of reactive oxygen species (ROS), is a symptom of the onset of the senescence [12,13].

*Prunus* species, as climacteric fruits, have a short ripening period and shelf-life after harvest, with a fast period of softening. The fruit firmness, soluble sugar content, and color changes are the parameters used to define the maturity stage, where a loss of firmness during ripening is the main limitation for apricot harvesting and commercialization. Hence, apricot fruit is often harvested unripe and still firm, stored at low temperature for some time and then moved to market to extend the supply period [5,14].

Fleshy fruits are enriched in flavor, mineral compounds, dietary fiber, vitamins, and antioxidants that make them an essential component of the human diet [15]. In this sense, fruit quality is a crucial factor for fruit consumption and acceptance by consumers, mainly due to the current situation of high competition in the markets with the presence of numerous new cultivars. In the case of *Prunus*, fruit quality is a complex human concept that includes sensory characteristics (including texture, taste, and aroma), aesthetic attributes (appearance, defect presence, sphericity, density, and firmness), and functional and nutritional values [16]. Consumers cherish the beauty and aromatic flavor of high-quality *Prunus* fruits, while other parameters, such as large fruit size, ease of handling, minimum processing, and long post-harvest shelf-life, are especially considered by the fruit industry. Therefore, the requirements by consumers and the priorities of *Prunus* breeding programs have led to the evolution of fruit quality parameters in the last few decades, increasing fruit taste with a good balance of sugars and acidity, improving attractiveness with orange flesh and reddish blush colors, and improving post-harvest behavior and shelf-lives [9,16]. From the point of view of the consumers, these traits increase the attractiveness and acceptance of new *Prunus* cultivars enriched in phenylpropanoids, carotenoids, and other nutraceutical compounds highly beneficial for human health [17,18,19,20,21].

At this moment, the level of information on the molecular switch that occurs during the transition from unripe to ripe fruits is limited. Increasing this knowledge will have important consequences from a breeding point of view. During fruit development, fruit becomes ripe due to changes in physiological structure and biochemical composition, resulting in unique aesthetic and fruit quality traits. The regulation of this process is affected by various internal factors (epigenetic developmental signals, hormone signaling, microRNA regulation) and external factors (light, temperature, nutrition, humidity, and biotic and abiotic stress) involving exchanges between the fruit and its environment. Therefore, fruit should be examined as an integrated system of biological process network [22]. Most *Prunus* fruit quality traits, such as sugar content, acidity, fruit color, firmness, and enrichment in nutraceutical and antioxidant components are controlled by a polygenic expression, quantitatively inherited, and influenced by environment stimuli and internal signaling grow regulators. From the genomic point of view, the variation of these traits has been traditionally studied following quantitative genetics approaches such as the correlation of genetic marker–trait association analysis and transcriptomic analysis for differential gene expression in order to elucidate the implied molecular mechanism. Hence, integrated phenotypic, metabolomic, genomic, and transcriptomic analysis of this complex process must be addressed because of the many applications in breeding that are oriented towards the increase of fruit quality traits related to the handling, processing, marketing, and consumption phases [23].

The objective of this review was to analyze the molecular bases of fruit quality in *Prunus* species through an integrated metabolic, genomic, and transcriptomic approach while also discussing the application of new biotechnology tools to *Prunus* breeding programs for fruit quality traits.

## 2. Regulation and Signal Transduction during the Ripening Process

The level of information of molecular events at the transcriptional, biochemical, hormonal, and metabolite levels underlying ripening in climacteric and non-climacteric fruits has considerably increased in recent decades. However, we still poorly understand the developmental switch that occurs in hormone responsiveness during the transition from unripe to ripe fruits [9]. One of the most critical factors in the ripening process is the developmental switch triggered by growth regulators, such as hormone response, that determine the transition from unripe to ripe fruits. Plant hormones are involved in the control of various aspects of fruit development throughout the different stages, organs, and tissues, thus constituting a complex network. Considering that every species, even cultivars, have different responses to hormones, it has been proven in *Prunus* species that the combined action of auxins, gibberellins, and cytokinins plays a major role in the regulation of fruit attributes; although ethylene and abscisic acid cause the main effects of fruit ripening in all the species [24,25,26,27].

### 2.1. Ethylene

Ethylene was the first known gas with biological significance as a signaling molecule for plant growth and development. Ethylene directly affects seed germination, cell elongation, cell differentiation, sex determination, fruit ripening, fruit senescence, and abscission [28]. Ethylene is also a fundamental regulator of responses to biotic stresses such as drought, flooding, wounding, chilling, pathogen infection, and chemical damage [29]. During fruit ripening, two ethylene-responsive elements are registered in immature fruit. In non-climacteric fruits, system I is responsible for basal rate ethylene production, which is slow, inhibited by exogenous ethylene (auto inhibition), and detected in vegetative tissues during growth processes. System II operates during ripening in climacteric fruits and is autocatalytic. The presence of two ethylene-responsive systems that differ between climacteric and non-climacteric fruits suggests that climacteric fruits evolved to synthesize ethylene in an autocatalytic manner as a positive feedback loop to control ethylene synthesis during ripening [30,31].

In both systems, ethylene is regulated through three enzymatic reactions: (a) the precursor S-adenosyl-L-methionine (AdoMet) is converted to S-adenosyl methionine (SAM) by S-adenosyl-L-methionine synthetase (MAT); (b) 1-aminocyclopropane-1-carboxylic acid (ACC) synthase (ACS) converts SAM to ACC; and (c) ACC oxidase (ACO) oxidizes ACC to release ethylene. The conversion of ACC to ethylene catalyzed by ACO is oxygen-dependent. However, under anaerobic conditions, ethylene formation is completely suppressed. Apart from ACC, ACS also produces 5′-methylthioadenosine (MTA), which is used for the synthesis of new methionine, thus ensuring high rates of ethylene biosynthesis [32,33,34,35,36].

Studies on the components of ethylene signaling have revealed a complex transduction pathway where the hormone is perceived by specifics receptors that initiate a transcriptional regulation cascade of genes underlying ripening-related traits including color, firmness, aroma, taste, and post-harvest shelf-life. Ethylene is perceived by ethylene receptor (ETR) and ethylene sensor (ERS) proteins. These receptors are negative regulators of ethylene signaling, and in the absence of ethylene, ERT activates the associated constitutive triple-response serine/threonine-protein kinase (CTR), acting as a negative regulator of the ethylene transduction pathway by suppressing the ethylene response via the inactivation of ethylene-insensitive (EIN) and ethylene-insensitive-like (EIL) proteins. The ethylene signaling cascade ends with the transcriptional activation of the transcription factors ethylene-responsive factors (ERFs), activated by EIN/EIL proteins. ERFs modulate the transcription of ethylene-regulated genes by binding to GCC-box type cis-elements present in their target promoters. It has also been suggested that the tetratricopeptide repeat (TPR) binds to ethylene receptors and leads to receptor degradation. These molecular regulators have been observed in different plant species including *Prunus* [32,33,36,37,38].

Ethylene is the main trigger of climacteric fruit ripening. Most biochemical changes associated with apricot ripening are under the control of ethylene [5]. A significant number of genes expressed during the transition of nectarine fruit from the preclimacteric to climacteric stages are not ethylene-regulated, but nevertheless a high upregulation of genes encoding transcription factors (TFs) belonging to several families like MADS-box, Auxine/indole acetid acid (IAA), basic leucine zipper domain (bZIP), basic helix–loop–helix (bHLH), homeodomain (HD )and myeloblastosis (Myb) [39]. In addition, treatments with the ethylene of unripe apricot fruit storage in cold conditions complete maturation by accelerating soluble solid accumulation, organic acid degradation, volatile organic compound (VOC) production, and fruit color change, thus leading to an improvement of apricot marketing [40,41,42]. CO_2_ is a competitive inhibitor of the ethylene effect, O_2_ is a substrate in the biosynthesis of ethylene, and ethylene oxide acts as an antagonist inhibitor of ethylene that delays the ripening process [28]. The compound 1-methylcyclopropane (1-MCP) can bind ethylene receptors as a competitive inhibitor that reduces ethylene action in fruit by suppressing the expression of genes related to fruit ripening, thus delaying the ripening process and prolonging storage life [41]. Aminoethoxyvinylglycine (AVG) acts as the most effective competitive inhibitor in the conversion of SAM to ACC, blocking ACS activity and reducing the rate of fruit softening in apricot during post-harvest with no effect in quality traits [43]. On the other hand, the application of ethephon, a long-lasting ethylene release compound, accelerates fruit ripening and abscission [44]. Therefore, there are multiple control levels of ethylene during ripening modulation [15].

### 2.2. Abscisic Acid

Abscisic acid (ABA) is an isoprenoid plant hormone. In higher plants, ABA is traditionally used to delay blooming, regulating seed dormancy and plant growth, and promoting ripening in climacteric and non-climacteric fruits [26]. ABA biosynthesis is derived from the oxidative cleavage of epoxy carotenoids like 9-cis-violaxanthin and 9-cis-neoxanthin by 9-cis-epoxycarotenoid dehydrogenase (NCED) to produce xanthoxin, the direct C15 ABA precursor [45]. In climacteric fruits, there is an accumulation of ABA content preceding climacteric ethylene production, reaching its maximum in fully ripe fruit and activating ethylene biosynthesis genes. A decrease of ABA was found to be correlated with an increase of ethylene, which is also related to a decrease of fruit firmness. In addition, a decrease of ABA is correlated with an increase in the sugar–acid ratio during the fruit ripening process. Thus, ABA may act as an original inducer for maturation in relation to ethylene action/perception [46]. Additionally, exogenous treatment with ABA accelerates the ripening process and increases the respiration rate when applied to pre-climacteric fruit but inhibits these processes when applied to post-climacteric fruit. The reduction of ABA content by biosynthetic inhibitors effectively delays maturity and softening [47].

### 2.3. Auxin

Auxins are compounds with an aromatic ring and a carboxylic group. Auxin modulates the response to light and gravity, general root and shoot architecture, organ patterning, vascular development, and plant growth. The effect of auxin differs between climacteric and non-climacteric fruits. Meanwhile, in climacteric fruits, auxin seems to accelerate the ripening process, and in non-climacteric fruit, auxin can negatively control the ripening [9,39]. In climacteric fruits like peaches, a concomitant increase of ethylene and auxin has been shown to exist. Instead, auxin plays an individual role during ripening, regulating the expression of different genes; thus, the hypothesis that cross-talk between auxin and ethylene existence has been supported [3].

### 2.4. Gibberellins

Gibberellins (GAs) are tetracyclic diterpenoids acids with an ent-gibberellane ring system. In apricots, the presence of GA1, GA3, GA5, GA6, GA8, GA29, and GA32 [48] has been described, and GA3 was found to be used in phytochemical treatments to induce the abscission of floral buds [49]. GAs play a stimulatory role in fruit development, enhancing cell division and enlargement, developing in parthenocarpic fruits when applied during fruit hardening, and delaying fruit ripening by decreasing ethylene release. This ethylene decrease is responsible for increasing fruit firmness, thus reducing susceptibility damage by mechanical compression and increasing time for fruit growth so that the fruit may reach a larger size. Gibberellin application during fruit set decreases flower bud populations for the following season, thus thinning the tree without phytotoxic effects, which may also increase fruit size due to the elimination of the competitive effect between developing fruits [50,51,52]. The application of GA in combination with ethephon, an ethylene long-lasting release compound used in agronomical treatments, can prevent the browning of pureed and sliced peaches [50]. On the hand, ethylene and ABA seem to be the antagonists of GA [53].

### 2.5. Cytokinins

Cytokinins (CKs) are N6-substituted adenine derivatives that play a crucial role during plant growth and development, including cell division, shoot initiation and growth, leaf senescence, apical dominance, sink/source relationships, nutrient uptake, phyllotaxis, vascular gametophyte and embryonic development, and responses to biotic and abiotic factors [54]. Endogenous levels of CKs induce fruit growth via the stimulation of cell division and could act by inhibiting auxin responses, at least partially, during fruit set and growth to increase fruit size [25].

### 2.6. Jasmonates

Jasmonates (JAs) are a class of oxylipins that induce a wide variety of higher-plant responses. In peaches, lipoxygenase (LOX), responsible for catalyzing the hydroperoxidation of polyunsaturated fatty acids to initiate the synthesis of oxylipins, was reported to increase at the early ripening stage and decrease in ripe fruit [55]. Using a peach as a model fruit regarding the effect of JAs during the ripening processes showed that exogenous JAs led to a ripening delay [56].

## 3. Determination of Ripening Date

The ripening date is an essential trait that defines the optimum harvesting moment. The ripening date is the moment of optimal physiological maturity when considering the conjunction of the main features of fruit quality. During ripening processes, hundreds of genes affecting firmness, skin and flesh color, sugar and acidity balance, and aroma are down- and up-regulated. The harvest date may vary from the ripening date because it is defined by growers based on a balance between high fruit quality, easy agricultural management, and the procurement of the greatest economic benefit. The selection of early or late harvesting varieties extends the marketed season by extending the production period. In this sense, the ripening date is one of the main *Prunus* breeding objectives, particularly for releasing extra-early ripening cultivars due to high market prices [3,5,57].

## 4. Fruit Color Development: Pigment Biosynthesis, Accumulation, and Degradation

Fruit color is one of the main external traits of apricot fruits perceived by consumers as an indicator of maturity, organoleptic quality, or attractiveness. During the ripening process, apricot skin color changes dramatically in a wide range from green to white, yellow, orange, or red, depending on the cultivar. This color change is due to the developmental transition from chloroplast to chromoplast as result of the degradation of chlorophyll and the dismantling of the photosynthetic apparatus, the biosynthesis of colored compounds like anthocyanins and carotenoids allow the accumulation/transformation of these compounds in different tissues or cellular components. The accumulation of pigments in fruits occurs during the ripening stage concomitantly with changes in firmness, sweetness, acidity, and aroma, and the distribution of pigments is subject to considerable variation between species and varieties [58].

During ripening and between maturity stages, apricot skin color changes dramatically from green to white, yellow, orange, red, or pink. The color change during fruit ripening is mainly due to the degradation of chlorophyll and the dismantling of the photosynthetic apparatus, the biosynthesis of different types of phenolic compounds like anthocyanins and carotenoids, and the later accumulation of carotenoid compounds in different tissues [3]. The red color is an attractive feature in apricot fruit and could have a great commercial impact, although this feature may be confusing regarding the optimal time of harvest [59]. Otherwise, the level of phenolic compounds is a key in food taste; it participates in the bitter, sweet, pungent, or astringent tastes of some products [60].

Though the flavonoid/anthocyanin biosynthetic pathway is well-understood, it is not clear how the initial precursors found in the cytosol are converted into the final pigment compounds, including β-cryptoxanthin, in the vacuole [58].

### 4.1. Anthocyanins

Anthocyanins are flavonoid compounds that are synthesized via the phenylpropanoid pathway. They are widely distributed secondary metabolites that play an essential role in skin pigmentation and antioxidant protection. These compounds are one of the best-characterized secondary metabolites, highly conserved among plants and microorganisms and providing a wide range of colors from orange/red to violet/blue. In *Prunus* fruits, an un-blushed skin color, which is green at the beginning that becomes red in conjunction with chlorophyll degradation and anthocyanin accumulation, seems to occur early in fruit development and maturation. In addition, the blushed side of apricot fruit corresponds to the sun-exposed side of the fruit. Additionally, different environmental conditions (year, location, and climate) appear to influence anthocyanin accumulation in apricots [59]. Thus, the development of a red blush in the skin is one of the most attractive features in apricot fruit with a tremendous commercial impact [61].

**Figure 3:**
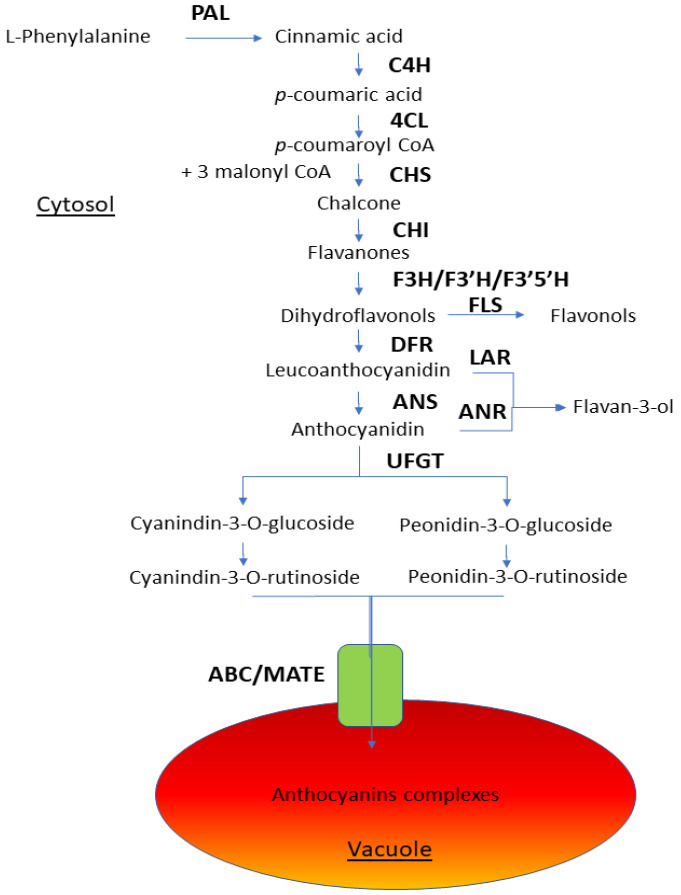
Anthocyanin biosynthesis pathway.

Initially the anthocyanin biosynthesis pathway stars from the key amino acid phenylalanine to produce 4-coumaryl Co-A by phenylalanine ammonia-lyase (PAL), cinnamic acid 4-hydroxylase (C4H), and 4 coumarate coenzyme A (CoA) ligase (4CL). In addition, the phenylpropanoid biosynthesis pathway stars from the amino acid L-phenylalanine to produce cinnamic acid by phenylalanine ammonia-lyase (PAL); cinnamic acid is transformed into p-coumaric acid by C4H, and then 4CL is added to p-coumaroyl CoA. Chalcone synthase (CHS) catalyzes the synthesis of chalcone, containing two phenyl rings, from one molecule of 4-coumaroyl CoA and three molecules of malonyl CoA, the principal precursors for all flavonoids. Chalcone is stereospecifically isomerized to the colorless flavanones by chalcone isomerase (CHI). Flavanones could be hydroxylated at different positions by flavonoid 3-hydroxylase (F3H), flavonoid 3′-hydroxylase (F3′H), or flavonoid 3′5′-hydroxylase (F3′5′H) to yield dihydroflavonols. Dihydroflavonol reductase (DFR) catalyzes the reduction from dihydroflavonols to leucoanthocyanidins, while flavonol synthase (FLS) leads to flavonols. CHS, CHI, F3H, F3′H, and F3′5′H are codified by early anthocyanin biosynthesis genes (EBGs). Leucoanthocyanin or anthocyanidin synthase (ANS) catalyze the synthesis of corresponding colored anthocyanidins. Anthocyanidins are initially 3-glycosylated by the action of uridine 5′-diphospho-glucuronosyltransferase (UDP) flavonoid 3-*O*-glucosyltransferase (UFGT), whose activity is required to catalyze the formation of stable glycosylated anthocyanins and is an important branching point enzyme that drives the pathway towards anthocyanins. In the absence of UFGT activity, the flux may be redirected towards other upstream flavonoid branches, such as the proanthocyanidin branch that yields flavonols. The late biosynthesis anthocyanins genes (LBGs) codify the enzymes of the last steps DFR, ANS, and UFGT. Flavonoid glycosides, including anthocyanins, after being synthesized in the cytosol, are usually transported into the vacuole, where they exhibit their brilliant colors. The transport mechanism of anthocyanins from the cytosol to the vacuole is facilitated by multidrug resistance and the toxic compound-like protein ATP-binding cassette transporter. Another transporter implicated in secondary metabolite transport is the multidrug and toxin efflux transporter (MATE) anthocyanin [62,63,64,65] (Figure 3).

The biosynthesis, accumulation, distribution, and degradation of anthocyanins in different plant tissues are determined by the expression pattern of structural and regulatory genes and environmental conditions. Anthocyanins are water-soluble, synthesized in the cytosol, and localized in the vacuoles of many flowers, leaves, fruits, seeds, and other tissues [58]. In peaches, apricots, plums, and grape berries, it has been shown that anthocyanins accumulate more in the skin than in the flesh [60,61,66,67]. In addition to modifications of their structures through the incorporation of different aromatic or aliphatic moieties, their specific color also depends on the environmental pH, co-existing colorless compounds (flavones and flavonols), and the presence of metal ions [58]. A total of 19 types of anthocyanins have been described, but six anthocyanins occur most frequently in plants: pelargonidin, cyanidin, peonidin, delphinidin, petunidin, and malvidin. The major compounds are cyanidin-3-*O*-rutinoside, cyanidin-3-*O*-glucoside, and peonidin-3-*O*-rutinoside [68,69]. Except for the last steps where the anthocyanins are decorated and translocated into the vacuole, anthocyanin biosynthesis is well-known [62,64,65,70,71].

The regulatory mechanism of anthocyanin biosynthesis is conservative but slightly different between species. The interaction between the R2R3- myeloblastosis (MYB), bHLH, and beta-transducin repeat (WD40) transcription factors (TFs), as well as their different combinations, in forming the MYB-bHLH-WD40 (MBW) ternary complex and joining gene promoters, determines the spatial and temporal expression of anthocyanin biosynthesis genes [62,72]. In apricots, environmental conditions, such as light and temperature, have been reported to have marked effects on anthocyanin biosynthesis and accumulation [59]. Anthocyanins are widely distributed secondary metabolites among plants that play important roles in the pigmentation of fruits. The pathway of anthocyanin biosynthesis is the best-characterized secondary metabolic pathway and is conserved among seed plants. Anthocyanins are flavonoids with a basic structure of C6-C3-C6 and the widest color range from pale yellow to red, purple, violet, and blue, depending on the molecule structure and vacuole pH. Flavonoids may be classified into six major groups, such as chaconnes, flavanones, flavonols, flavandiols, anthocyanins, and condensed tannins (or proanthocyanidins); a seventh group, the aurones, is widespread but not ubiquitous. Some plants also synthesize specialized forms of flavonoids, such as the isoflovonoids that are found in legumes and small number of non-legumes plants [73]. Anthocyanins are produced by a specific branch of the flavonoid pathway. Flavonoid biosynthesis genes are highly conserved at the structural and functional levels among species and are organized in different branches that lead to the production different flavonoids. Some branches are species-specific, whereas others are almost ubiquitous. Each branch is controlled by similar R2R3-MYB regulatory genes in combination or not with bHLH and WD40-type regulatory genes, whereas in most species, the anthocyanin branch is controlled by a ternary complex of MYB-bHLH-WD40 TFs. A well-known physiological functions of the anthocyanin pigments and flavonol co-pigments include the recruitment of pollinators and seed dispersers, the signaling between-plant-and-microbe defense as antimicrobial agents, and the UV protection. Six anthocyanidins occur most frequently in plants: pelargonidin, cyaniding, peonidin, delphinidin, petunidin, and malvidin. The sugars commonly linked to anthocyanidins are monosaccharides (glucose, galactose, rhamnose, and arabinose) and di- or tri-saccharides formed by the combination of the four monosaccharides. Moreover, many anthocyanins have sugar residues acylated with aromatic or aliphatic acids [73].

The biosynthesis, accumulation, distribution, and degradation of anthocyanins in different plant tissues are regulated by genetic and environmental conditions, and these factors finally determine the expression pattern of the structural and regulatory genes of the metabolic pathway [72]. In *Prunus* species, anthocyanin accumulation is responsible for yellow and red coloration in plums [65,74], apricots [59], peaches [63], and sweet cherries [75]. In addition, anthocyanins provide a wide range of colors ranging from orange/red to violet/blue. A total of 19 types of anthocyanins, aglycons, or chromophores of anthocyanins are known at this moment, but there are only six major ones widely present in fruits: pelargonidin, cyaniding, peonidin, delphinidin, petunidin, and malvidin. Anthocyanins are modified by glycosyl moieties in versatile ways in a family- or species-specific manner. From the biochemical point of view, anthocyanins are most frequently O-glycosylated at the C3-position, followed by the C5-position. The glycosylation of anthocyanins results in a slight reddening. The glycosyl moieties of anthocyanins are commonly modified by aromatic and/or aliphatic acyl moieties. Aromatic acylation causes a blue shift and stabilizes anthocyanins in many plant species [58].

In peaches, the highest accumulation of anthocyanins has been found to be around the stone in the fruit mesocarp. Anthocyanin concentration increases in the skin, reaches a maximum and then decreases toward the end of maturation. There are three possible explanations for the anthocyanin decrease in the skin of mature fruits. The first involves the degradation of anthocyanins, molecules which are known to be unstable in weakly acidic conditions. The second explanation involves a dilution effect induced by fruit growth due to fruit growth being faster than anthocyanin biosynthesis. Thirdly, peeling becomes difficult as fruit ripening progresses, thus yielding two consequences: the inclusion of flesh particles in skin preparation and a longer duration of skin preparation that allows for the increased oxidation of polyphenols. In *Prunus* species, CHS, F3H, DFR, and UFGT, as well as localized acquired resistance (LAR) and activator of anaerobic (ANR), are expressed at higher levels in the peel and around the stone and at lower levels, sometimes at background levels, in the mesocarp. The transcript levels of genes encoding enzymes at the top (CHS) and end (UFGT) of the anthocyanin pathway are much higher than those of other biosynthetic genes. A positive correlation between gene expression and anthocyanin content was found for F2H, DFR, and UFGT [63]. Additionally, a correlation between the expression of the anthocyanin biosynthetic UFGT and the anthocyanin concentration in the peel at the early and late stages of fruit development was reported [64], although their transcript level was extremely low [63].

In the peach genome, three main *MYB10*-*like* and three *bHLH*-*like* TFs have been identified as being linked to anthocyanin biosynthesis including *MYB10.1* (ppa026640m), *MYB10.2* (ppa016711m), and *MYB10.3* (ppa020385m) located within 80 kb on linkage group (LG) 3, as well as within the two closest DNA markers (CC2 and CC12A) to the *Ag* trait [63]. The maximum expression of all three genes is found in the flower, while in the mesocarp, expression during ripening was relevant only for *MYB10.1* and *MYB10.3*. Three peach *bHLH* TFs are also involved in the regulation of flavonoid biosynthesis (*bHLH3*, ppa002884m; *bHLH33*, ppa002645m; and *GL3*, ppa002762m). However, only *bHLH3* has been found to increase during fruit ripening. Nonetheless *bHLH3*, with *bHLH33* and *GL3*, is also expressed during fruit growth, possibly participating in the control of the synthesis of other flavonoids or in other developmental programs, such the JA response pathway. Finally, a third regulator, a WD40-repeat protein, is involved in the regulation of anthocyanins biosynthesis. A peach WD40-repeat protein (ppa08187m) is expressed in high levels during ripening; however, this constitutive regulator is only present as a single copy in peach genome [63].

In apricot fruits, the un-blushed skin color, green at the beginning, becomes red in conjunction with chlorophyll degradation and anthocyanin accumulation, which seems to occur early in fruit development and maturation. In addition, the blushed side of apricot fruits corresponds to the sun-exposed side of the fruit. Additionally, different situations (year, location, and climate) appear to influence anthocyanin accumulation in apricot. The major compound in apricot fruit skin is cyanidin-3-*O*-rutinoside, followed by cyaniding-3-*O*-glucoside, and peonidin-3-*O*-rutinoside [59]. Apricot species present the largest variability in fruit color of *Prunus* species, ranging from white (‘Moniqui’) and orange (‘Goldrich’) to fruits largely covered with a strong red blush such as ‘Orange Red’. Intervarietal crosses have been performed between different contrasted cultivars, and recombinant hybrids have been obtained to study their physical and physiological behavior together with the genomic expression of their target genes [42,59,61,76,77,78].

In sweet cherry, 13 candidate genes that are responsible for anthocyanins in fruit have been identified from differentially expressed genes (DEGs) between red and yellow sweet cherry varieties during the ripening process. The genes belonging to anthocyanins biosynthesis pathway are PAL, 4CL, CHS, CHI, F3H, F3′H, DFR, ANS, and UFGT. The expression of these 13 genes exhibits distinctive patterns in the two varieties. In red sweet cherry, all the DEGs encoding anthocyanin biosynthesis shows a significantly upregulated expression during the ripening process, particularly when the fruit turns red (S3 and S4) [75]. In contrast, all the DEGs encoding anthocyanin biosynthesis in fruit of a yellow cultivar show significantly downregulated expression during the beginning of the ripening process (S1 and S2) and remain at a low expression level at the end (S3 and S4). The expression levels of PAL, CHS, and F3′H in the red fruit are lower than those of the yellow fruit at S1. However, in S3 and S4, the expression levels of PAL, 4CL, CHS, CHI, F3H, DFR, F3′H, ANS, and UFGT in the red fruit are higher than those of the yellow fruit. This indicates that the biosynthesis of anthocyanin compounds are maintained at high levels in the red cultivar when the fruit is turning red at the end of the ripening process. However, there are no significant changes in the expression of UFGT in a yellow cultivar, and a gradual increase expression of this gene is correlated with the accumulation of anthocyanidins in the red cultivar; therefore, UFGT may play a key role in sweet cherry coloration [75].

In the case of the Japanese plum, TFs of the subfamily *R2R3MYB* of the *MYB* gene family are associated with the regulation of anthocyanin biosynthesis. Analyses have shown that a sustained increase in the expression of *PsMYB10* begins in S2 (Figure 2) in the skin of all red cultivars, and it continues until S4, showing the highest positive correlation with anthocyanin accumulation and *LDOX* and *UFGT* gene expression. These results suggest a putative function of *PsMYB10* in the regulation of the transcriptional control during anthocyanin biosynthesis. On the other hand, there is a significant negative correlation between anthocyanin accumulation, *LDOX* and *UFGT* gene expression, and the highest expression of *PsMYB1* at all yellow tissues. These genes participate in the synthesis of proanthocyanidins, which are abundant in S1 step. Late genes *UFGT* and *LDOX* show expression peaks in the S3 and S4 stages in the skin of Japanese plum red cultivars. All tissues without anthocyanin accumulation present a minimal expression of both *LDOX* and *UFGT* at all development stages, while pigmented tissues have an increased expression of them, suggesting that both genes are expressed in a coordinated manner during changes of fruit color. However, a major correlation between the amount of anthocyanins and gene expression in skin and pulp was found with *LDOX* expression. This evidence suggested that *CHS* is the key regulation point in the flavonoid pathway, which participates in the incorporation of phenolic compounds to the biosynthesis of proanthocyanidins and anthocyanins, whereas *LDOX* gene expression and its regulation could be crucial for anthocyanin biosynthesis during fruit development in this species [64,65].

The modification of the sequence of TFs may affect the regulation of anthocyanin biosynthesis. In red grape cultivars, anthocyanin biosynthesis is controlled by a complex genetic locus with two MYB genes (VvMYBBA1 and VvMYBA2) that regulate UFGT and the last steps of the anthocyanin pathway. These genes are inactive in some white grape cultivars due to a retrotransposon insertion in the promoter region of VvMYBA1 and non-conservative substitutions in the coding region of VvMYBA2 [79]. In addition, the existence of a link between the regulatory genes controlling fruit ripening and the down-stream anthocyanin pathway has been described. A MADS-box TF, VmTDR4 from bilberry (*Vaccinium myrtillus* L.) appears to control anthocyanin accumulation during fruit ripening via the direct or indirect control of the expression of the R2R3-MYB gene VmMYB2. When VmTDR4 is silenced by virus-induced gene silencing, the expression of VmMYB2, CHS, and other anthocyanin-specific biosynthesis genes (DFR and ANS) is reduced and two-to-three fold less anthocyanins are produced [80].

Finally, the influence of agro-climatic and environmental conditions has been reported to have a marked effect on anthocyanin biosynthesis and accumulation. Anthocyanin biosynthesis is enhanced by light, and MYB TFs appear to be the primary determinants of fruit pigmentation in response to light in red apples [81]. In red apricots, agro-climatic conditions control anthocyanin biosynthesis. The stability of the red skin character was studied over two successive years on two apricot cultivars located in different orchards, and they were found to display significant differences in anthocyanin concentration between years. The significant differences may reflect differences of climate and cultural practices among locations, including differences in light radiation, temperature, water stress, and/or mineral nutrient availability [73]. Temperature and, in particular, the difference between day and night temperatures have been reported to have a marked effect on anthocyanin accumulation in apples, berries, plums, grapes, and pomegranates [60]. On the other hand, in apple fruit skin, ultraviolet-B (UV-B) radiation and low temperature are important factors for anthocyanin accumulation because they induce the expression of the anthocyanin biosynthetic genes, especially CHS, ANS, and UFGlut, an analogue to UDP-glucose [82].

### 4.2. Flavonoids

In plants, flavonoids are synthetized in the cytosol. The biosynthetic enzymes belong to various enzymes families, such as 2-oxoglutarate-dependent dioxygenase (OGD), cytochromes P450 (P450), and glucosyltransferases (GT), which suggests that plants recruit theses enzymes from pre-existing metabolic pathways. The EBGs are chalcone synthase, chalcone isomerase, flavanone 3-hydroxylase, flavonoid 3′-hydroxylase, flavonoid 3′5′-hydroxylase, and dihydroflavonol 4-reductase, which lead to the production of flavonols [58]. The LBGs, including *anthocyanidin synthase/leucoanthocyanidin dioxygenase*, *anthocyanidin reductase*, *leucoanthocyanidin reductase* and *UDP flavonoid 3*-*O*-*glucosyl transferase*, lead to the production of proanthocyanidins in seeds and anthocyanins in vegetative tissues. On the other hand, CHS, a polyketide synthase, is the first enzyme in the pathway and catalyzes the synthesis of narengine chalcone (THC), a chalcone containing two phenyl rings from one molecule of 4-coumaroyl CoA and three molecules of malonyl CoA, the main precursors for all flavonoids. In addition, (2S)-naringenin is hydroxylated at the 3-position by flavanone 3-hydroxylase (F3H) to yield (2R,3R)-dihydrokaempferol. F3H belongs to the OGD family, yielding dihydroflavonols. F3H also catalyzes the hydroxylation of eriodyctiol and pentahydroxyl flavanones to dihydroquercetin and dihydromyricetin, respectively. Flavonoid 3′-hydroxylase (F3′H) and flavonoid 3′,5′-hydroxylase (F3′5′H), which are P450 enzymes, catalyze the hydroxylation of dihydrokaempferol (DHK) to form (2R,3R)-dihydroquercetin and dihydromyricetin, respectively. F3′H and F3′5′H determine the hydroxylation pattern of the B-ring of flavonoids and anthocyanins, which are necessary for leucocyanidin and leucodelphinidin production, respectively. Initially, dihydroflavonol 4-reductase (DFR) catalyzes the reduction from dihydriflavonols to leucoanthocyanidins and ANS (also called leucoanthocyanidin dioxygenase—LDOX), which belongs to the OGD family, catalyzes the synthesis of corresponding colored anthocyanidins; this ends via the activity of the UFGT glycosylation of anthocyanidins, which yields anthocyanins [62,63,65].

Different UDP-glycose-dependent glycosyltransferases, including glycosyltransferases family 1 and acyltransferases (ATs) mainly belonging to the BAHD superfamily of enzymedand S-adenosylmethionine-dependent methyltransferases, have been indicated as responsible for the anthocyanidin diversity. These enzymes are specific to the position of modification on the anthocyanin and donor substrates. These anthocyanidins are initially 3-glucosylated by the action of UFGT [58]. In addition, UFGT activity is required to catalyze the formation of stable glycosylated anthocyanins. In the absence of UFGT activity, however, the flux may be redirected towards other upstream flavonoid branches, such as the pro-anthocyanidins [62].

The expression of flavonoid biosynthesis genes in most vegetable species correlates with anthocyanin synthesis, but there is some variability in the specific involved step. In most species, only LBGs correlate well with anthocyanin synthesis, such as in tomato [83] and pepper [84]. However, in other species, the transcript levels of not only LGBs but also some EBGs are higher in red compared to non-red fruits, such the Chinese bayberry [85] and apples [81]. In *Prunus* species, studies based on RNA-Seq ordered to characterized the role of structural and regulatory genes in the flavonoid pathway in different tissues and development stage have been performed on cherries [75], peaches [63], nectarines [64], and Japanese plums [65]. Flavonoid glycosides, including anthocyanins, after being synthetized in the cytosol are usually transported into the vacuole, where they exhibit their brilliant colors. The transport mechanism is less well-understood than biosynthesis, which may be redundant or depend on plant species and organs. The first and most established mechanism involves the transport of anthocyanins via a glutathione S-transferase (GTS)-like protein and multidrug resistance and toxic compound-like protein ATP-binding cassette transporter [a type of ATP-binding cassette (ABC) transporter]. The molecular mechanism through which these proteins, especially GSTs, achieve the transport has not yet been clarified [58].

It has been shown that in the Rosaceae family (including *Prunus*), CHS, CHI, and F3H are early genes and have a coordinated expression pattern that reaches its peak in the first stage of fruit development. The late genes LDOX and UFGT have their peak of maximum expression in the maturation stage, especially in tissues that accumulate anthocyanin. The TFs MYB10 and MYB1 have been implicated in the regulation of the last three enzymes of the metabolic pathway, DFR, LDOX, and UFGT, that are the key to explain contrasting patterns of anthocyanin accumulation [65].

### 4.3. Carotenoids

Carotenoids are the most widespread group of colorful pigments found in plants, algae, fungi, and bacteria. Carotenoids serve as accessory pigments to harvest light in the photosynthetic apparatus, have photoprotective functions during photosynthesis, participate in root–mycorrhizal interactions, and attract pollinating insects and animals that contribute to seed dispersal in fruits and the provision of substrates for abscisic acid biosynthesis. Additionally, apocarotenoids or norisoprenoids result from cleavage carotenoids, yielding aromatic compounds [42,58,86]. Carotenoids are responsible for yellow/orange skin ground color and flesh color in apricot fruits. In apricots, a relationship between skin and flesh color with carotenoid content was demonstrated [76,87], and a non-destructive method to determine the content of carotenoids, β-carotene, and pro-vitamin A based on the flesh and skin color of the edible portion developed [77]. Carotenoids supplied to the human diet have important health benefits such as protective activity against several human cancers, cardiovascular diseases, and degenerative diseases [88,89,90,91,92,93,94].

**Figure 4:**
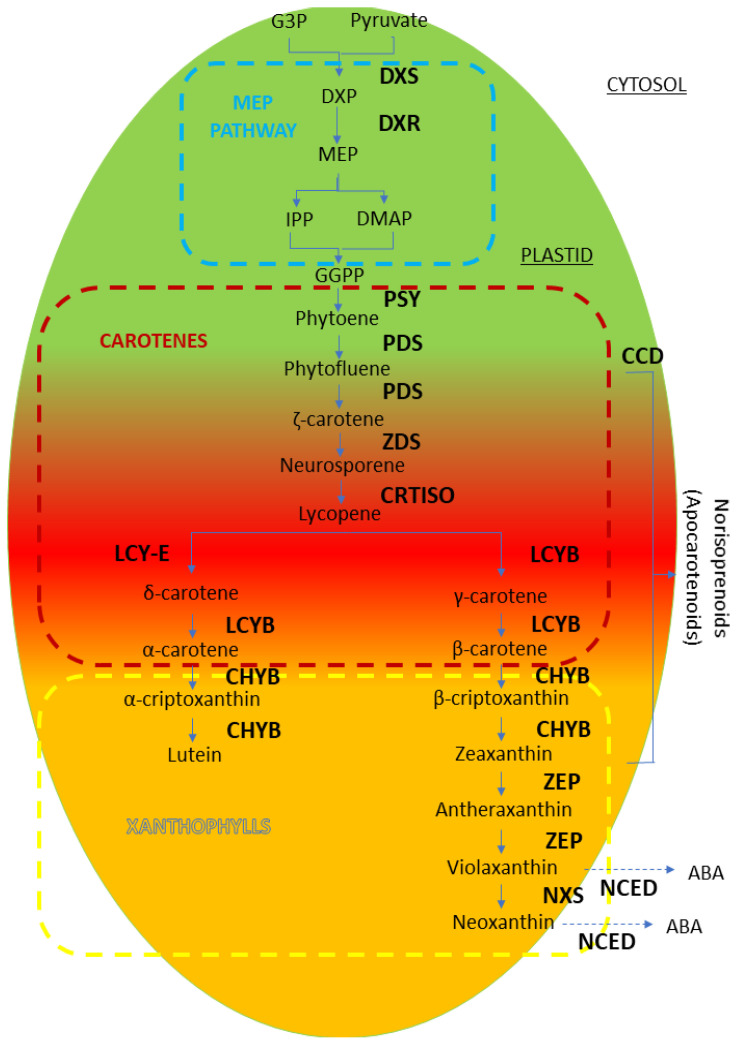
Carotenoid biosynthesis pathway.

Carotenoid biosynthesis is a well-defined pathway composed of a series of desaturation, cyclization, hydroxylation, and epoxidation steps (Figure 4). Carotenoids are accumulated during the ripening process, producing changes in skin ground color and flesh color in fruit. Usually, carotenoid accumulation confers yellow-to-red coloration to fruits. Carotenoids such as zeaxanthin, violaxanthin, antheraxanthin, and lutein are invariably found in leaves and stems. On the other hand, carotenoids in non-green tissues show distinctive compositions depending on the species [95,96,97,98,99,100].

Carotenoids are a subclass of terpenoid (or isoprenoid) compounds (C_40_) with polyene chains that may contain up to 15 conjugated double bonds. They play essential roles in plant life, e.g., photoprotective functions during photosynthesis as components of photosystems and the provision of substrates for the biosynthesis of the plant growth regulator ABA. Carotenoid accumulation is a balance between synthesis and degradation. However, during fruit ripening, the accumulation of carotenoids largely results from an increased synthesis capacity of chloroplasts and chromoplasts. In apricot fruits, β-carotene is the major carotenoid pigment, followed by β-cryptoxanthin and γ-carotene [87].

In carotenoid biosynthesis (Figure 4), the 2-C-methyl-d-erythritol 4-phosphate (MEP) pathway starts with the condensation of glyceraldehyde 3-phosphate (G3P) and pyruvate by the enzyme 1-deoxy-d-xylulose-5-phosphate synthase (DXS), yielding 1-deoxy-d-xylulose-5-phosphate, which is reduced by 1-deoxy-d-xylulose-5-phosphate reductoisomerase (DXR) and generates MEP; this is followed by several steps until the levels of isopentenyl pyrophosphate (IPP) and dimethylallyl pyrophosphate (DMAPP) rise. The carotenoid biosynthesis pathway starts from the C5 IPP in plastids, and it is there that the product accumulates. Three IPPs and one DMAPP are condensed to form C_20_ geranylgeranyl pyrophosphate (GGPP). A head-to-head coupling of two GGPP molecules, catalyzed by phytoene synthase (PSY), yields the first C_40_ carotenoid, phytoene. Then, conjugated double bonds are added by phytoene desaturase (PDS) and ζ-carotene desaturase (ZDS), yielding the intermediate pigments phytofluene (colorless), ζ-carotene (pale yellow), neurosporene (orange–yellow), and lycopene (red), containing 5, 7, 9, and 11 conjugated double bonds, respectively. The cyclization of lycopene is a branch point in the pathway that is catalyzed by lycopene β-cyclase (LCYB) and lycopene ε-cyclase (LCYE), producing two types of carotenes that contain one or two rings of either the β- or ε-type. When only LCYB acts in this step, lycopene is converted to β-carotene and β-cryptoxanthin, and it is then further metabolized to zeaxanthin via two hydroxylation steps by carotene β-hydroxylase (CHYB), providing a variety of oxygenated derivates called xanthophylls. Epoxidation at positions C5,6 and C5′,6′ of the β-ring of zeaxanthin, catalyzed by zeaxanthin epoxidase (ZEP), yields antheraxanthin and violaxanthin then metabolizes to neoxanthin by neoxanthin synthase (NXS). Both 9-cis-violaxanthin and 9-cis-neoxanthin are cleaved to xanthoxin (C_15_) by NCED and then converted to ABA via an ABA aldehyde intermediate. Carotenoid cleavage dioxygenase (CCD) is involved in the degradation of carotenoids at multiple levels, yielding apocarotenoid compounds [86] (Figure 4).

Carotenoids, which are isoprenoid compounds (C_40_) with polyene chains containing up to 15 conjugated double bonds, are found ubiquitously in all plants and microorganisms. They play essential roles in plant life, including photoprotective functions during photosynthesis as components of photosystems and the provision of substrates for biosynthesis of the plant growth regulator ABA. Carotenoids also play an important role in human nutrition and health, providing provitamins with anti-cancer activities [92] and cardiovascular protection effects [94]. In addition, carotenogenesis in plant tissues is predominantly regulated at the transcriptional level [101]. The successful isolation of different genes for carotenoid biosynthesis will allow for the identification of the key regulatory steps of carotenoid biosynthesis. Nevertheless, knowledge on the molecular aspects that regulate the pathway is still limited. Recently, the genes responsible for *hp1* and *hp2* (mutations conferring a high level of carotenoids) have been shown to encode the proteins UV-Damaged DNA-Binding Protein 1 (DDB1) and Deetiolated 1 (DET1), components that are widely involved in the light-signal transduction pathway [102].

Carotenoids confer yellow-to-red coloration to flowers and fruits, showing qualitative differences depending on the plant organs and species. Carotenoids such as zeaxanthin, violaxanthin, antheraxanthin, and lutein are invariably found in leaves and stems. In contrast, carotenoids in non-green tissues show distinctive compositions that depend on the plant species. The main carotenoids of the flower petals of most plants are yellowish xanthophylls that are pale-to-deep-yellow in color. The petals of some plants have a modified carotenoid biosynthesis capacity, accumulate unique carotenoids associated with their respective genus or even species, and are orange-to-red in color [58]. In apricot fruits, β-carotene participates in the fruit coloration together with anthocyanins. The orange color conferred by β-carotene is masked by anthocyanins in the case of red cultivars [59].

On the other hand, in plants, the entire pathway starting from a C_5_ isoprene, IPP, occurs in the plastids, and it is there that the product accumulates. Four IPPs are condensed to form C_20_ GGPP. A head-to-head coupling of two GGPP molecules, catalyzed by PSY, yields the first C_40_ carotenoid, phytoene. In tomatoes, two different types of PSYs (Psy-1 and Psy-2) are expressed in an organ-specific manner [99]. *Psy*-*1* encodes a fruit- and flower-specific isoform and is responsible for carotenogenesis in chromoplasts. In green tissues, Psy-2, which is homologous to *Psy*-*1* but highly divergent from it, is predominantly expressed and makes a major contribution to carotenogenesis in chloroplasts. Conjugated double bonds are subsequently added by two structurally similar enzymes, PDS and ZDS. These desaturation reactions yield the intermediates phytofluene, ζ-carotene, neurosporene, and lycopene that contain 5, 7, 9, and 11 conjugated double bonds, respectively. Increasing the number of conjugated double bonds shifts the absorption to longer wavelengths, resulting in colorless phytoene and phytofluene, pale-yellow ζ-carotene, orange–yellow neurosporene, and red lycopene. During the desaturation steps, several intermediate reactions with a cis-configuration to form all-trans-lycopene is carried out by carotenoid isomerase (CRTISO). The cyclization of lycopene is also a branch point in the pathway, catalyzed by lycopene β-cyclase (LCYB) and lycopene ε-cyclase (LCYE). Because LCYE adds only one ε-ring in most plants, the pathway typically proceeds only along branches, leading to carotenoids with one β-ring and one ε-ring (α-carotene and its derivatives) or two β-rings (β-carotene and its derivatives). β- and α-carotenes are further modified by hydroxylation or epoxidation, providing a variety of structural features. The hydroxylation of the β- and ε-rings is catalyzed by β-hydroxylase (CHYB) and ε-hydroxylase (CHYE), respectively [58]. In addition, epoxidation at positions C5,6 and C5′,6′ of the β-ring of zeaxanthin, catalyzed by ZEP, yields violaxanthin synthase (NSY). Both 9-cis-violaxanthin and 9-cis-neoxanthin are cleaved to xanthoxin (C_15_) by NCED and then converted to ABA via ABA aldehyde intermediate [100].

The amount of carotenoids in fruit tissues is not solely attributed to the ability to synthesize carotenoids. The mechanism that controls carotenoid accumulation is largely unknown. Recently, two different regulatory mechanisms were postulated: carotenoid degradation, and sink capacity [58] through a gene encoding carotenoids cleavage dioxygenase (CCD4). A transcriptomic analysis during the ripening process in peach cv. Fantasia by µPEACH1.0 showed an increase of a homolog of β-carotene hydroxylase that is responsible for the hydroxylation of β-carotene to β-cryptoxanthin [103].

### 4.4. Chlorophylls and Photosynthetic Apparatus

Though the fruit is not a photosynthetic organ, it contains chlorophyll (Chl), which is responsible for the characteristic green color of fruits when they are still immature, from the beginning of its development. Chl breakdown occurs in fruit tissues during the ripening process, causing a rapid degreasing and consequently unmasking the existence of pigments like carotenoids or anthocyanins [104].

The coordination of Chl breakdown is an integral process of maturation that is developmentally programmed. The pheophorbide A oxygenase (PAO)/phyllobilin pathway has been described to be the major Chl catabolic pathway that is highly conserved in different plant tissues and species. The pathway involves two distinct phases: the early phase deals with the degradation of phototoxic free Chl molecules in the chloroplast, and the late phase is responsible for modifications of colorless Chl catabolites and their translocation from the chloroplast to the vacuole. Chl catabolites are now termed phyllobilins, known as non-fluorescent catabolites of Chl (NCCs), and they have an effective natural antioxidant activity in ripening fruits that may suggest a further physiological role helping to inhibit the decline of vital functions in plants and possess beneficial properties for human health [105,106,107].

In a fruit, the chlorophyll degradation pathway initially involves the conversion of chlorophyll b (Chl b) to chlorophyll a (Chl a) by a hydroxymethyl chlorophyll b reductase (HCAR). Then, two different dephytylation enzymes catabolize Chl a, chlorophyllase (CLH), and pheophytin pheophorbide hydrolase or pheophytinase (PPH) to transform pheophytin to pheophorbide. Later, the Fe-dependent oxygenase PAO is responsible for transformed pheophorbide to red chlorophyll catabolites (RCCs). RCCs are then reduced to primary fluorescent chlorophyll catabolites (pFCC) by RCC reductase (RCCR) to NCCs, which are the final product [104].

## 5. Biochemical Pathways Related to Flavor

Most *Prunus* fruits have a unique flavor derived from the combination of taste and aroma; as such, flavor serves as an indicator of nutritional value and attractiveness for consumers, thus playing a central role in fruit quality and consumer’s acceptance. The taste of apricot primarily depends on an increase in sugars and organic acids and the degradation of bitter principles as flavonoids, tannins, and related compounds. Meanwhile, the aroma depends on a complex mixture of VOCs, which increase and accumulate during fruit ripening in the peel and contribute to the production of a complex mixture of compounds in the fully ripe stage. Flavor compound accumulation patterns and concentration differs between tissues, species, and even cultivars. The contents of aroma volatiles and organic acids are generally more abundant in the skin than in the flesh, controlled by a combination of regulatory networks triggered by hormone signaling, developmental factors, and stress responses [108].

### 5.1. Aroma

VOCs are generally released during the ripening process through the catabolism of high-molecular-weight compounds like proteins, carbohydrates, and fatty acids. VOC production is highly regulated under different conditions and developmental stages [109]. Mediated by ethylene, there are several VOC biosynthetic pathways such as fatty acid metabolism, amino acid metabolism, ester biosynthesis, and carbohydrate metabolism. The aroma of fruits is influenced by pre-harvest factors like the genotype, growing conditions, and maturity stage. Post-harvest factors, like storage temperature, atmosphere, ethylene control, and wax coatings, also affect aroma [110].

In *Prunus*, the fruity odor compounds significantly increase during fruit development, while the green odor compounds rapidly decrease. A total of 46 aroma compounds have been identified in *Prunus* fruits, including eight aldehydes, five alcohols, seven esters, five norisoprenoids, eight lactones, ten terpenes, and six acids [108,109,110]. Among them, 18 aroma compounds are present in an apricot fruit’s aroma and significantly change during fruit ripening, including three aldehydes (hexenal, (Z)-3 hexenal, and (E,Z)-2,6-nonadienal), three apocarotenoids (β-damascenone, β-ionone, and dihydro-β-ionone), five lactones (γ-octalactone, δ-octalactone, γ-decalactone, δ-decalactone, and γ-dodecalactone), five terpenes (β-myrcene, linalool, α-terpineol, geraniol, and limonene), and two esters (hexyl acetate and (Z)-3-hexenyl acetate) [14]. From these compounds, β-ionone and γ-decalactone are responsible for the characteristic apricot aroma. On the whole, VOC metabolism in the skin is significantly higher than in the flesh, where the contents of total aldehydes and terpenes decrease during fruit development and ripening, thus releasing aroma compounds [14]. Terpenes are the predominant group of VOCs that originate from carbohydrate metabolism. As primary metabolites, terpenes are derived from carotenoids and then form a group of volatiles terpenes: apocarotenoids or norisoprenoids. The most abundant aroma compound in apricots is the apocarotenoid, where β-ionone represents 90% of the total identified apocarotenoids [108]. Saturated and unsaturated fatty acids are the major secondary precursors for VOCs in apricot fruits, and they are responsible for the synthesis of lactones, the second most abundant aroma compounds. Lactones rapidly increase during ripening, where γ-decalactone is the predominant lactone compound in apricot [108,109,110].

In apricot fruits, odor compounds such as esters will increase significantly during late fruit development (Figure 2), while the green color compounds such as hexenal rapidly decrease in this late step. A total of 46 aroma compounds, including eight aldehydes, five alcohols, seven esters, five norisoprenoids, eight lactones, ten terpenes, and six acids, have been identified. Among them, 18 aroma compounds, including three aldehydes (hexenal, (Z)-3 hexenal, and (E,Z)-2,6-nonadienal), three apocarotenoids (β-damascenone, β-ionone, and dihydro-β-ionone), five lactones (γ-octalactone, δ-octalactone, γ-decalactone, δ-decalactone, and γ-dodecalactone), five terpenes (β-myrcene, linalool, α-terpineol, geraniol, and limonene) and two esters (hexyl acetate and (Z)-3-hexenyl acetate) are the major compounds in apricots. γ-decalactone is the major compound, followed by β-ionone and γ-dodecalactone The total aldehydes significantly decrease during the development of apricot fruits [14]. However, the most abundant aroma compounds are β-damascenone, β-ionone, and dihydro-β-ionone, which dramatically increase during fruit ripening, especially the major apocarotenoid β-ionone. On the other hand, lactones are the second most abundant aroma compounds that rapidly increase during the ripening process, and γ-decalactone is the predominant lactone. Additionally, two major esters, hexyl acetate and (Z)-3-hexenyl acetate, increase during the entire development and ripening period. The contents of total aldehydes and terpenes decrease rapidly during fruit development and ripening, while the contents of total lactones and apocarotenoids keep increasing during the same period. On the whole, peels present significantly more individual or total aroma volatiles than pulps [14].

In apricots, a significant increase of LOX, hydroperoxide lyase (HPL), alcohol dehydrogenase (ADH), alcohol acyl-transferases (AAT), acyl-CoA oxidase (ACX) activities have been observed during the different steps (Figure 2) of the development of apricot fruits. A rapid significant increase in CCD activity has been found, whereas terpene synthase (TPS) activity decreases significantly during this process. Regarding the fatty acid pathways, the unsaturated fatty acids linoleic acid (18:2) and linolenic acid (18:3) can be cleaved into hydroperoxides by LOX and then subsequently cleaved by HPL to form hexanal or nanonal compounds. Aldehydes can then be reduced to the corresponding alcohols by ADH. AAT catalyzes the final linkage of an acyl moiety and an alcohol to form esters [111]. In *Prunus* fruits, the β-oxidation of fatty acids is also considered another pathway for lactone formation because the first enzyme acyl-CoA oxidase of this process is involved in lactone formation [14]. The accumulation of lactones, especially for γ-decalactone, keeps consistent with ACX enzyme activity increases and provides further evidence for β-oxidation being involved in lactone formation. Carotenoids can be cleaved into volatile apocarotenoids in fruit by CCD. The increase in CCD enzyme activity correlates with the accumulation of apocarotenoids during fruit ripening. In addition, light-colored cultivars contain abundant β-ionone and dihydro-β-ionone because the carotenoid in these cultivars may be cleaved into volatile apocarotenoids, suggesting that CCD is important for the conformation of color and aroma quality. Furthermore, the contents of β-ionone, β-damascenone, and γ-decalactone suggest that white apricots present stronger flowery and peach-like aromas than other cultivars. TPS is considered the key enzyme for volatile monoterpenes, which is consistent with the decrease found throughout the development and ripening processes. Thus, TPS may also be involved in monoterpene biosynthesis. In general, the enzyme activities of aroma volatile metabolism in peels are significantly higher than those in pulps, suggesting a higher aroma volatile synthesis [14].

### 5.2. Taste

Taste development is due to an increase in sweetness, a decrease in acidity, and the accumulation of secondary metabolites like phenylpropanoids and tannins until achieving a well-balanced sugar/acid content and the degradation of astringent or bitter compounds. Taste evolved as a dynamic trait during fruit ripening due to increased gluconeogenesis, the hydrolysis of polysaccharides, decreased acidity, and the accumulation of sugars and organic acids [3]. In most fruits, fructose and glucose are the most important portions of soluble sugars, reaching the peak of maximum concentration at maturation or during ripening. For organic acids, malic acid and citric acid are quantitatively predominant, usually accumulate at the early stages of fruit development, and are used as respiratory substrates during fruit ripening, decreasing their concentration at the end [14]. In general, the composition of sugars and acids in fruits mainly depends on genetics; however, cultivation conditions and environmental factors may influence the final total contents. Desirable taste depends on the consumer’s preferences, trending to sweeter taste with a pinch of acid, a complete loss of astringent taste, and an intense aroma, reaching a well-balanced sweetness/acidity at physiological ripening [16].

#### 5.2.1. Soluble Solids

In *Prunus* fruits, all sugars including fructose, glucose, sucrose, and total sugar increase rapidly throughout the entire development process. In all cases, from the turning of the pulp/peel color to the full-ripe stage, the sugar contents increase steeply. However, no significant differences of sugar content during the early development have been found. The difference of sugar content between the peel and the pulp may dependent on the cultivar [3]. Glucose is the predominant sugar during early development and its ratio significantly decreases in parallel to the total sugar content during ripening. The second major sugar during development is fructose, which follows the same concentration pattern as glucose. The ratio of sucrose increases during development, becoming the major sugar during ripening. Finally, at the late stage of fruit development, the content of sucrose exceeds that of glucose. *Prunus* fruits mainly accumulate glucose during early development, whereas sucrose is mainly accumulated during ripening. All these results suggest that sugar accumulation in fruits varies from glucose-predominant to sucrose-predominant during development and ripening [3,14].

For example, in apricot fruits, nine enzymes involved in sugar metabolism have been analyzed in fruits during the entire development process. Regarding the enzymes involved in sucrose accumulation, a significant increase of sucrose synthase (SS) activity responsible for synthesis direction (SSthy) and sucrose phosphate synthase (SPS) is found throughout development and is responsible for the rapid increase of sucrose in the late ripening stage. In addition, a significant increase of activity in SS responsible for degradation direction (SSca) is observed during the ripening process. Neutral invertase (NI) and acid invertase (AI), which cleave sucrose, do not significant change the expression during ripening. On the other hand, sorbitol can be inverted into fructose by sorbitol dehydrogenase (SDH), and a significant increase SDH enzyme activity has been observed during the entire development period. Fructose can be cleaved by fructokinase (FK), but no significant change has been found after this cleavage. In addition, sorbitol can be oxidized into glucose by sorbitol oxidase (SO), and a significant increase is observed during the fruit development period. In contrast, glucose can be cleaved by glucokinase (GK), but no significant change is observed in the enzyme activity. Therefore, it is possible that sorbitol is converted to glucose and fructose via SO and SDH, which suggests that the accumulation of these sugars mainly comes from sorbitol catalysis. Finally, we can conclude that SS, SO, and SDH might play an important role in sugar accumulation and are under tight developmental control in apricot fruits, although no significant differences in these enzymes have been observed between peels and pulps, thus indicating a coordinated ripening process in fruits, including mesocarps and exocarps [14].

#### 5.2.2. Acidity Loss

During the development of a *Prunus* fruit, there is a continuous accumulation of organic acids, and their final concentration is determined by the balance between the biosynthesis of organic acid, its degradation, and its vacuolar storage [112,113]. Almost all organic acids, including oxalate, tartrate, quinate, malate, citrate, fumarate, and total organic acids, increase during early development and then decrease rapidly during apricot fruit ripening. Malate, citrate, and quinate occupy around of 95% of total organic acids in both the skin and flesh at the end of ripening [14], where malate represents 80% of total organic acids presents in apricot at the beginning of the ripening process and decreases with time [108]. At the early stages of fruit development, malate and quinate are the predominant compounds accumulated, and they are used as respiratory substrates during fruit ripening. Meanwhile, the ratio between organic acids changes with the rapid increase of citrate at the ripening stage, followed by a small decrease of quinate and malate. The significant decrease in citrate and the small reduction of malate contribute to the acidity loss of ripe fruit. Oxalate and tartrate are found at lower levels, while traces of fumarate are also detected during the development period [108,113].

The biosynthesis of malate requires the fixation of CO_2_ on a carbon skeleton derived from hexose catabolism, which is achieved by phosphoenolpyruvate (PEP), catalyzed by PEP carboxylase (PEPC). In plants, cytosolic PEPC is necessary for the synthesis of tricarboxylic acid (TCA) cycle acids from sugars. PEPC catalyzes the conversion of PEP to oxaloacetate (OAA). Then, OAA is converted to malate by cytosolic nicotinamide adenine dinucleotide (NAD)-dependent malate dehydrogenase (NAD-MDH), and malate is transported across the tonoplast into the vacuole, where is stored. MDH catalyzes a reversible reaction between OAA and malate, helping to balance the concentration of these two metabolites. The malic enzyme (ME) catalyzes the reversible conversion between malate and pyruvate. Recently, it has been suggested that the conversion of malate to pyruvate is under the control of fumarate. When fumarate accumulates, the conversion of malate to pyruvate is facilitated [114]. Once malate and OAA have been synthesized in the cytosol, they can be converted into tricarboxylates, mostly citrate, or other dicarboxylates through two metabolic pathways—the TCA cycle and the glyoxylate cycle. In turn, citrate can be converted into dicarboxylate via several pathways (TCA cycle, glyoxylate cycle, γ-aminobutyrate (GABA) shunt, and acetyl-CoA catabolism). All these conversion reactions modify the acidity of fruit cells [113]. In plants, the color of anthocyanins changes depending on the vacuolar pH, co-existing colorless compounds (co-pigments—typically flavones and flavonols), and metal ions. The regulation of the vacuolar pH, however, which greatly affects anthocyanin color, is only partly understood. The only known structural genes that regulate vacuolar pH with relevance to color is the Japanese morning glory (*Ipomea nil*) antiporter and the *petunia PH4* gene, which activates vacuolar acidification in relation to the *R2R3 Myb* gene [58].

In peach fruits, the final organic acid concentration in the ripe fruit is determined by the balance of organic acid biosynthesis, degradation, and vacuolar storage. During fruit development, apricot undergoes a continuous accumulation of organic acids, which are used as respiratory substrates [112]. All organic acids including oxalate, tartrate, quinate, malate, citrate, fumarate, and total organic acids are mostly increased during the early stages of fruit development and decrease until fruits were full-ripe. Quinate, malate, and citrate are the predominant organic acids throughout fruit development and the ripening period. Additionally, oxalate and tartrate are found at lowers levels, while trace fumarate is also detected during the development period. Regarding the ratio of whole organic acids, malate is the first major organic acid in apricots, and the ratio of malate in fruit decreases during development. The ratio of quinate also decreases during development and ripening. However, the ratio of citrate significantly increases during development. Quinate and malate are the major organic acids at the early stage of development and ripening (Figure 2), whereas the ratio changes with the rapid increase of citrate at the maturation stage. Quinate, malate, and citrate occupy around of 95% of total organic acids in both peels and pulps at the end of ripening [14].

In apricot fruits, six enzymes involved in organic acid metabolism have been identified during fruit development and ripening. In this species, quinate dehydrogenase (QH) is responsible for converting 3-dehydroquinate into quinate, the last step for quinate synthesis, and a significant decrease is found in apricot during fruit development and the ripening process. The decrease of quinate and QH suggest that QH is the key enzyme for quinate accumulation. In addition, malate synthase (MS) is the key enzyme for synthesis, and a significant decrease in its activity is observed throughout the development process. Malate can also be metabolized by the nicotinamide adenine dinucleotide phosphate (NADP-malic enzyme (NADP-ME) and the NAD-malic enzyme (NAD-ME). The decrease in MS and increase in NADP-ME and NAD-ME are together responsible for the malate decrease throughout the development and ripening processes in this species, which further confirms the correlation between their enzymatic activity and malate accumulation. On the other hand, citrate synthase (CS) is mainly responsible for citrate biosynthesis, and its activity is significantly increased during fruit development and ripening. It has been reported that glutamate decarboxylase (GAD) activity participates in regulating cytosolic pH, while no significant change in GAD activity is found in apricots throughout the development process. Citrate also can be metabolized in the GABA shunt pathway by GAD, but the increase in CS without an increase of GAD indicates that the GABA shunt is not important for citrate accumulation in apricot fruits. No significant differences in the six enzymes for organic acid metabolism have been observed between peels and pulps, but significantly higher levels of citrate are found in pulps than in peels. Even though citrate is not the sourest of the organic acids, citrate gives a stronger taste than quinate and malate, which may strengthen the integration of sourness taste of apricot pulps [14].

### 5.3. Cell Wall Degradation and Texture

During fruit ripening, fleshy fruits undergo textural changes generally based on the premise that cell wall polysaccharide breakdown leads to a loss of tissue firmness and ripening-associated softening. Texture change is interdependently coordinated by a wide range of cell-wall enzymes acting on both the middle lamella and the primary cell wall that, together with alteration of the turgor pressure, cause the weakening of the cell wall structure and thus lead to seed dispersal and the final conversion of unripe hard fruit into edible soft and crispy fruit [115,116]. This softening is largely responsible for the post-harvest lifespan, an increase of susceptibility to pathogens, and a lower tolerance to mechanical damage that may quickly render the fruit unmarketable [3]. This implies a short-shelf-life, which is a great limitation to the transportation and storage of fresh apricots due to the rapid flesh softening. Softening is cultivar-dependent and highly influenced by maturity stage at harvest, storage conditions such as temperature and relative humidity, and storage duration [117].

In order to reduce fruit loss after harvest, *Prunus* fruits are usually harvested at early maturity; storing at low temperature, decreasing O_2_ and increasing CO_2_ in the atmosphere, and treatments with 1-MCP are efficient methods to avoid softening [118,119,120]. Physiological disorders, such as juiciness loss, mealiness formation (woolliness), or flesh browning, happen when apricots are removed from a long cold storage to room temperature [121]. Fruit texture can influence general consumer appreciation. Texture is defined as a result of the combination of complex factors at different scales. It involves cell turgor pressure, cuticle composition and structure, cells size, shape, and distribution in different tissues. Texture encompasses several different descriptors, such as firmness (defined as resistance to compression) and juiciness (perceived by consumers and evaluated by a trained jury); however, it is difficult to assess instrumentally [122]. Cell wall disruption joined to the internal turgor pressure generates an expanding sound pressure wave perceived by the human senses as crispness. This feature, related to the integrity and rigidity of the cell wall, is also associated with freshness as juice release from fruit [123].

Fruit texture is assessed by measuring the force required to compress or penetrate the fruit surface. The resistance of fruit to compression is affected by intercellular adhesion, shear forces between polymers, and cellular turgor pressure that forces the membranes of individual cells against the cell walls [116]. Fruit firmness deeply affects fruit quality. In addition to the fact that consumers prefer crunchy and juicy fruits, firmer flesh helps in the harvesting, handling, and shipping of fruits before their consumption [103].

Plant primary cell walls are composed of cellulose micro fibrils and matrix substances like pectin polysaccharides and hemicelluloses. It is assumed that the biochemical properties of cell walls change substantially during ripening, becoming weaker or thoroughly degraded and rebuilt. The modification of cellulose/hemicellulose and pectin polymer networks contribute to cell wall strength and tissue integrity, and they are believed to underline changes in firmness and texture, but the type and magnitude of the alterations carried out during ripening may vary considerably, thus leading different textures associated with ripe fruit, even within cultivars of the same species [3].

The only way to understand the importance of fruit cell wall metabolism is to consider the modification of the whole polymer network rather than individual polymers. The standard approach to elucidate fruit softening is based on the identification of wall components whose solubility increases and polymer size decreases in parallel with decreasing fruit firmness; this identification aids the characterization of proteins that are expressed during ripening correlated to the observed wall changes [116].

Within the context of fruit ripening, pectin solubilization, depolymerization, demethylesterification, hemicellulose depolymerization, and neutral sugar loss are some common ripening-related cell wall modifications [3]. From this point of view, wall restructuring is a synergistic process involving the simultaneous interaction of numerous families of cell wall modifying proteins, like expansin (EXP), endoglucanases (EG), xyloglucan endotransglycosylase-hydrolase (XTH), pectin methylesterases (PME), β-galactosidase (βGAL), pectin lyase (PL), and polygalacturonase (PG). The recycling of the sugars released as a consequence of cell wall turnover indicates that the primary cell wall is not only a sink for polysaccharide components but also a dynamic structure exhibiting the long-term reorganization and degradation of specific polymers during development [124,125,126,127,128,129].

Plant enzymes that hydrolyze cellulose and hemicellulose in polysaccharide networks are referred to as endoglucanases and are expressed at high levels during the ripening process. Expansins are cell wall proteins with a wall-loosening activity that contributes to cell expansion, wall disassembly, and fruit texture. Expansins are associated with numerous tissues and developmental stages undergoing changes in size and shape in ripening fruits [130]; they are accumulated abundantly from the immature green stage to the half-ripe stage of fruit development and decrease after that, displaying a positive correlation with fruit size. Additionally, a cinnamyl alcohol dehydrogenase (CAD) facilitates the conversion of p-hydroxycinnamaldehyde into the monomeric precursors of lignin and is considered to play a role in the strengthening of the cell wall structure [131].

As described previously, during the *Prunus* fruit ripening process, the middle lamella dissolves, cells collapse, and cell walls break down due to pectin and hemicelluloses depolymerization and solubilization, reducing polymer length, branch amounts, and aggregate sizes [132]. The increase in water-soluble pectin and the decrease in insoluble pectin methyl-esterification and cell wall galactose content lead to the loss of cohesion of pectin gel matrix, cell wall dissolution, and cell separation by the combined action of PG, PME, and β-GAL in the middle lamella. As a consequence, these changes affect the calcium-mediated cell–cell adhesion and contribute to softening. EXP induces plant cell enlargement by disrupting non-covalent linkages such as hydrogen bonds between cellulose and hemicellulose without any cell wall hydrolytic activity, and it seems to be down-regulated by ethylene in apricot fruits [120,132,133,134,135].

In apricot fruits, softening begins largely before the climacteric crisis [5] and it is really fast during development and ripening [14]. Fruit firmness, couple with the color change of the skin, is the parameter used to define the apricot maturity stage that is decreased during ripening.

In peaches, the expression of genes involved in softening stars before the appearance of the ethylene climacteric rise at the S3 stage, and the occurrence of the ethylene climacteric rise can be deduced by following the expression of PpACO-1 [136]. The modification of cell wall is believed to underline changes in firmness and texture, but the type and magnitude of the alterations carried out during ripening may vary considerably between fruit species, thus leading to different textures associated with ripe fruit, even within cultivars of the same specie (e.g., melting, non-melting, and stony hard peaches). Pectin solubilization, depolymerization, demethylesterification, hemicellulose depolymerization, and neutral sugar loss are some common ripening-related cell wall modifications [3].

## 6. Nutraceuticals and Antioxidant Compounds

There are several epidemiologic pieces of evidence on how daily fruit consumption has beneficial health effects [137]. In addition to the traditional quality traits related to aesthetic or flavor, fruit consumers also value other attributes like nutraceutical availability and phytochemical compounds with beneficial health effects. Fresh apricot fruits are an excellent source of precursors for vitamin A (carotenoids), antioxidant compounds (polyphenols, flavonoids, and anthocyanins), and fiber [138]. Numerous compounds (carotenoids, phenylpropanoids, and dietary fiber) present in fresh apricot have potentially anticarcinogenic effects [139,140]. Dried apricot is particularly rich in minerals like calcium and iron [138].

Fruit and vegetables are natural sources of carotenoids as animals are unable to synthesize carotenoids de novo and rely upon their diet. There is considerable interest in dietary carotenoids because they play an essential role in human nutrition and health due to their high antioxidant potential, provision of provitamin A, alleviation of age-related diseases [99], display of anti-cancer activity [92], cardiovascular protection effects [94], and anti-amyloidogenic activity against Alzheimer’s disease [141]. The major carotenoid compound found in apricot fruit is β-carotene [61], with antioxidant properties that may protect against free radical damage. β-carotene can be metabolized to vitamin A, inhibits cell proliferation, and helps in the differentiation of healthy epithelial cells [142]. In addition, the precursors for carotenoid terpene compounds induce the production of enzymes that deactivate carcinogens, prevent carcinogens from reacting with target sites, and possibly prevent hormones that promote tumor growth [139]. Other carotenoid compounds found in apricot ripe fruit (but in small amounts) are phytoene, phytofluene, γ-carotene, lycopene, β-cryptoxanthin, and lutein [143]. In our study, only β-carotene and β-cryptoxanthin were considered as carotenoids health promoters because provitamin A carotenoids include β-carotene, β-cryptoxanthin, and α-carotene [87]. β-cryptoxanthin is a chemo-preventive agent for lung cancer [144], can prevent osteoporosis [145], and reduces the risk of developing inflammatory disorders [146]. The carotenoids phytoene and phytofluene should be of no antioxidative significance, and the other compounds are found in such small concentrations that their effects on human health are not considered [147].

On the other hand, phenolic compounds are some of the main sources of antioxidant activity in apricot fruits [17,148,149,150]. The main phenolic compounds present in apricot are gallic acid, chlorogenic acid, neochlorogenic acid, caffeic acid, p-coumaric acid, ferulic acid, (+)-catechin (−)-epicatechin, quercetin-3-galactoside, quercetin-3-glucoside, quercetin-3-rutinoside, and kaempferol-3-rutinoside [151]. In apricots, the contents of phenolic compounds differ between the skin and the flesh, always being higher in the skin [61]. In *Prunus* species, the main flavonol compound is routine (plus traces of quercetin 3-hexoside), whereas kaempferol rhamnosyl-hexoside (plus quercetin-acetyl-hexoside) is always present in smaller amounts. The main phenolic compounds are chlorogenic acid and neochlorogenic acid. Finally, anthocyanin pigments are only found in small amounts in apricot varieties with a red blush in the skin, and cyanidin-3-*O*-glucoside and cyanidin-3-*O*-rutinoside are the major compounds [59,61]. Flavonoids, like quercetin and kaempferol, are polyphenolic compounds with potent antioxidant activity that may reduce cell proliferation, extend the action of vitamin C, inhibit clot formation, and have anti-inflammatory effects. Chlorogenic acid has been described in the in phenol compounds and can prevent cancer-causing nitrosamines; in addition, caffeic acid prevents the formation of carcinogens and blocks the reaction of carcinogens with cells, coumarin increases the activity of glutathione transferase (a detoxification enzyme), and catechin has potent antioxidant activity. Additionally, anthocyanins are powerful antioxidants and beneficial to human health against coronary diseases and cancer [149].

Finally, *Prunus* fruits are a good source of dietary fiber. Dried apricots contain less fiber than fresh apricots, and fruit peeling decreases the fiber content [152]. Dietary fiber has been described to be protective against colon cancer due to the capacity to bind carcinogens, increase fecal bulk, and decrease intestinal transit time, as well as its ability to be fermented by intestinal microflora in the colon, leading butyrate, which has been shown to be antineoplastic [153,154,155].

## 7. Application of New Molecular Tools to *Prunus* Breeding and Selection for Fruit Quality

The achievement of high fruit quality is one of the main objectives proposed in breeding programs. The ripening process is an essential step and the final stage during fruit development that gives rise to fruit with fruit quality traits due to physiological differentiation and biochemical compound accumulation. All these changes are the results of a coordinated modulation of a gene expression network regulated by complex and interrelated mechanisms affected by internal and external factors. Enhanced quality fruit are the main goal of plant breeding due the increasing interest in functional foods, new varieties, and, in particular, foods rich in antioxidants like anthocyanins and carotenoids, with a bigger size, with early or late ripening stages, with better flavor and texture, with attractive colors, and with other traits that positively influence a consumer’s buying decisions. At the molecular level, the identification of transcription factors, key genes of biosynthesis pathways, and triggering environmental effectors that control the traits are related. However, the application of new biotechnologies at the molecular level to the final integration of genomic, transcriptomic, metabolomic, and phenotypic analyses will provide a global view of the entire process focused on improved fruit quality in apricot breeding programs [103,156]. Future challenges will consist of unravelling the molecular mechanisms underlying plant development and fruit ripening. The identification of TFs, key genes of biosynthetic pathways, and triggering environmental effectors controlling the expression of fruit quality traits and their applications to develop specific molecular markers for using marker-assisted selection (MAS) in apricot breeding programs will improve the efficiency of releasing new, high-quality, fruit cultivars [23,157].

In this context, high-throughput sequencing (HTS) technologies have undergone significant progress in recent years due to the emergence of new and improved sequencing technologies and the development of bioinformatics tools that allow for great availability, low cost, efficiency, timeliness, comprehensive analyses, accurate results, and less data errors. The advance in HTS technology also generated computational challenges in the way the data are deposited, formatted, and remain accessible for use by the scientific community. As a future perspective, recent third-generation genomics technologies like long-read sequencing developed by PacBio and optical mapping bring the opportunity to improve the quality of sequencing experiments. Meanwhile, the application of machine learning algorithms in bioinformatics tools and higher computation capacities allows for the filling of gaps and the enabling of low-cost solutions for genomic and transcriptomic research. As a result of the drastic decrease in the cost of sequencing over the last few years, up to 450 plant genomes are now available (3 October 2019, http://www.ncbi.nlm.nih.gov). From these genomes, 93 are assembled and annotated with Kyoto Encyclopedia of Genes and Genomes (KEGG), KEGG orthology Genome (KOG), ENZYME, Pathway and InterPro (Phytozome v12.1.6, http://phytozome.jgi.doe.gov), including the most important commercial crops. The low cost of this technology has greatly accelerated the implementation of HTS in projects focused on omics science research, thus significantly expanding the scope of studied species.

Over the last 15 years, the Genome Data Base for Rosaceae (GDR) (https://www.rosaceae.org/) has been enhanced with new genomic, genetic, transcriptomic, and phenotypic data. High quality reference genomes of main species from the *Prunus* genus—including *Prunus persica* whole-genome assembly v1.0, v2.0, and annotation v2.1 (v2.0.a1) [158,159], considered as the reference *Prunus* genome; *Prunus mume* L. genome v1 [160]; *Prunus avium* whole-genome assembly v1.0 and annotation v1 (v1.0.a1) [161]; *Cerasus* × *yedoensis* ‘Somei-Yoshino’ genome v1.0 assembly and annotation [162]; *Prunus domestica* draft genome assembly v1.0 and annotation v1.0.a1 [163]; *Prunus dulcis* ‘Lauranne’ genome v1.0 [164]; *Prunus dulcis* ‘Texas’ Genome v2.0 [165]; *Prunus armeniaca* V1 [166]; and *Prunus salicina* v1.0 [167]—are publicly available. The development of these complete genomes will allow for a precise reference of the obtained molecular results and the development of high-throughput methods for genomic analysis involving the most abundant genetic variation (single nucleotide polymorphisms: SNPs) and transcriptomic analysis at the gene expression level (DEGs) [23,168].

### 7.1. Genomics

Since the introduction of DNA markers, linkage analysis has become an essential tool in genomics research. Quantitative traits, often called complex or polygenic traits in contrast to simple Mendelian traits or monogenic, are traits where the genotype–phenotype relationship cannot be directly observed. A quantitative trait locus (QTL) is a genomic region responsible for quantitative genetic variation of a trait where the allelic variation of a locus and the variation of the trait is associated. On the other hand, a Mendelian trait locus (MTL) is described as a genomic region linked to a unique gene responsible for a trait. Genetic mapping and QTL identification are usually the first approaches for the identification of candidate genes linked to characters of agronomic interest that could enable the development of molecular markers for early MAS applied to the optimization of *Prunus* breeding programs. For this purpose, segregating progenies are used for molecular characterization, the construction of genetic linkage maps, and the establishment of a relationship with agronomic traits by genetic linkage maps and trait loci analysis, including QTLs and MTLs [169].

Genetic linkage map construction and QTL identification require genotyping with numerous DNA markers distributed throughout an entire genome. Before HTS technology became available, genetic maps had a low density and were constructed using only a few markers and small populations due to the high cost of genotyping. This approach resulted in the detection of a large amount of QTLs containing a high number of putative candidate genes, making their identification and functional validation difficult. Recent advances in computational infrastructure and bioinformatics analysis have created many new possibilities to effectively deal with complex data. The increased amount of genomic resources, such as whole-genome sequencing and high-density genotyping platforms, has enabled QTL analysis to be more readily converted to markers that can be directly applied to MAS applied to fruit quality breeding [170].

In apricots, QTLs linked to fruit quality traits have been identified, mainly in LGs 3 and 4 in apricots [171,172,173,174,175]. These LGs have been also identified as key genome regions for fruit quality traits in other *Prunus* species including almonds [176], peaches [177,178,179,180,181], and Japanese plums [170,182]. The most significant QTLs are located in LG4 for soluble solid contents and ripening dates, as well as in LG3 for skin ground and flesh color in different analyzed *Prunus* species [183,184,185,186]. Flesh color around the stone, flower color, and other color have been mapped to LG 3, fruit skin color and leaf color have been mapped to LG6, blood flesh has been mapped to the top of LG4, and dominant blood flesh has been mapped to the top of LG5 [63,187,188]. Regarding ground color, Socquet-Juglard et al. [175] reported that when scored as a qualitative trait, the *Sc* locus for red or green color could be mapped in peaches to LG6 [187,189], and QTLs with a low repeatability over two years of observation were mapped on LG3, LG6, and LG7 by Eduardo et al. [178]. Meanwhile, Dirlewanger et al. [189] did not find any QTLs for skin color. In apricots, Salazar et al. [173] identified QTLs on LG2, LG3, and LG6 for ground color. Additionally, complementary studies along *Prunus* species will provide stable QTLs and will also be used to identify candidate genes within the QTLs. It has been described that some QTLs are stable between *Prunus* species, and they have similar trends in their QTL effects [187,190]. In addition, efforts are underway to use high-density molecular markers and to anchor loci to consolidate this information into a single genetic consensus or reference map for each species [191]. More recently, the first QTLs linked to phytochemical compounds were identified [192].

### 7.2. Transcriptomics

The initial transcriptome studies relied on hybridization-based microarray technologies that had a limited ability to identify and quantify a great diversity of molecules and a wide range of RNA expression levels. Later, due to the development of HTS technologies, RNA-Seq has become a powerful tool for transcriptomics analysis [193]. The µPEACH1.0 microarray has been used in comparative studies between apricots and peaches, showing a significant hybridization (≈43% of spotted probes). Following this, a transcriptome profile analysis during fruit development that was carried out separately showed that 71% of genes had the same expression pattern in both species [31,194,195]. The limitation of this technology is the restriction of only evaluating the probes that are printed in the microarray, the impossibility of discovering new transcripts not yet described, and the loss of information in samples with a low hybridization. However, more recently, the method of choice for the deep prospection of the transcriptome by the scientific community has become the RNA-Seq due to the combination of the discovery and quantification of gene expression in a single high-throughput sequencing assay [196]. Additionally, behind the significant advance, research in plant transcriptomics has shown numerous unresolved problems related to the alignment and assembly of sequences, gene annotation, the identification of alternative splicing sites, SNP discovery, and downstream analysis for the quantification of transcription such as DEGs, the identification of isoforms, the identification of fusion genes (chimeras), the identification of involved metabolic pathways, DEG clustering, and the construction of co-expression networks [197].

However, the development of HTS, the remarkable advances in computational theory and bioinformatic algorithms for data analysis, and the availability of public databases have facilitated the study of gene expression in transcriptome analysis. The correlation between genomic, transcriptomic, metabolomic, and phenotypic expression may allow us to elucidate the mechanism of genetic regulation expression and the key genes involved in the main metabolic pathways altered during the ripening process. The identification of those candidate genes differentially expressed in genotypes that differ in fruit quality traits could be useful for the prospection of their regulatory mechanisms and the presence of genomic variants (e.g., allelic variants, indels, duplications, inversions, mutations, and transposable elements). The identification of candidate genes or genomic variants linked to the expression of agronomical traits of interest will allow for the development of specific molecular markers that could be used in MAS to improve the efficiency of breeding programs. The ripening process has been greatly studied from a transcriptome perspective by using RNA-Seq in many other related species from the *Prunus* genus like apricots [198,199,200], peaches [111,201,202,203,204,205,206,207], Japanese plums [74,208,209], Japanese apricots (*P. mume* L.) [210,211], and cherries [75,212]. These transcriptomic findings should be very useful to develop RNA markers to monitor the ripening process. In addition, the validation of major genes localized in QTL through gene expression analysis using qPCR (expressed QTLs—eQTLs) has recently been assayed in different *Prunus* species including peaches [213] and apricots [171]. eQTLs are then derived from polymorphisms in the genome that result in differential measurable transcript levels, where a correlation between genotype and expression levels can be detected [169]. These associations can unravel the genetic basis of complexes traits with a composite of genes interacting, as in the case of fruit color and fruit sorbitol content [214,215,216].

### 7.3. Epigenetic Regulation

Epigenetic changes are chemical modifications affecting DNA or structural DNA proteins (called histones) within the chromatin. Two main types of epigenetic modifications have been described: DNA methylation (in plants, usually 5′-cytosine methylation) and posttranslational histone modifications including the acetylation and methylation of histones (H2A, H2B, H3, and H4) [217,218]. DNA methylation associated with cell status stability and regulation mainly occurs in three sequence contexts: CG and CHG, which are found in promoter and coding regions, and CHH (where H = A, C or T), which is found in non-coding regions and transposable elements (TEs) [219]. A genome-wide analysis of DNA methylation by bisulfite sequencing consisting of the high-throughput sequencing of bisulfite-treated DNA samples (epi-GBS) is the most extended methodology to evaluate DNA methylation marks. This epi-GBS technique has been developed as a cost-effective exploration and comparative analysis of DNA methylation. In addition, this methodology can be used in the DNA methylation analysis without a reference genome [220].

Recent studies have linked epigenetic evidence with the ripening and production of anthocyanins in *Prunus*. Cheng et al. [221] addressed the link between the genetic program of fruit ripening and DNA methylation state, ultimately obtaining the full peach methylome sequences of different stages of fruit development from immature to ripe. More recently, Lü et al. [8] showed additional evidence regarding the role of epigenetic regulations in fruit ripening. The authors analyzed DNA methylation and histone post-translational modifications PTMs landscape in the frame. The authors characterized the fruit epigenome of more than 12 fruit species including peaches. In this species, the authors showed that the histone mark H3K27me3 seemed to be conserved in marking genes involved in the ethylene biosynthesis. The authors hypothesized that the mechanisms involved in fruit flesh might arise from dry fruit species. All this evidence suggests that the epigenetic regulation of fruit characters happens within the *Prunus* species. Comparative DNA methylation studies will surely contribute to our knowledge of methylation variants and provide candidate epialleles linked to agronomic traits including fruit quality traits. Such polymorphisms can be screened in large populations using NGS (next generation sequencing) to confirm the locus methylation state associated with a given character of interest [219].

### 7.4. Marker-Assisted Selection for Superior Fruit Quality Genotypes

*Prunus* species are characterized by a juvenile period of at least three years and long generation cycles; hence, the ability to eliminate undesirable seedlings from progenies in breeding populations through MAS reduces cost and allows breeders to focus on populations comprised of individuals carrying desirable alleles of genes of interest [191]. When the genes involved in fruit quality traits are not known, the phenotype could be predicted based on the allelic variants of the molecular markers flanking the previously identified QTLs or MTLs [169]. If the trait is simply inherited, the expression of the gene of interest is recessive, or the evaluation of the character is otherwise tricky, MAS could be applied early in the developmental stage, is economical, and has a high probability of success in comparison with traditional methods [222]. Then, undesirable genotypes could be eliminated from progeny populations using MAS in early developmental stages when fruit quality traits have not yet been expressed. Therefore, the final goal of the identification these QTL regions is to identify expressed major genes insidethese QTL regions, to convert conventional QTL into expression QTL (eQTL) in order to biologically validate the QTLs responsible for the analyzed traits in segregating genotypes [223]. For QTL or MTL validation, the effectiveness association of the DNA marker–phenotype should be checked. DNA markers applied to MAS will provide a substantially improved probability of selecting superior genotypes compared with conventional breeding and evaluation practices, and so they can be used as a part of a reliable and robust technique in routine genotype selection. In apricots, most of the QTLs identified to date are inconsistent for use in MAS due to the influence of statistical tests, the low contribution to the genetic variation of the trait, and the differential gene expression of the trait depending on the year (year by year variability) [222].

On the other hand, regarding the identification of candidate genes to MAS development, in peaches, several major genes have been cloned or fine mapped and are postulated as strong candidate genes applied to MAS linked to fruit quality traits [224]: *NAC TF* for ripening date in peaches [225], cell number regulator (CNR) for fruit size in peaches and cherries [226,227], *leucine*-*rich repeat receptor*-*like kinase* for fruit shape [228], *cleavage carotenoid dioxygenase* (CCD) for flesh color (white/yellow) [95,229], *MYB TFs* for flesh and skin color in peaches [230,231], *NAC TFs* for blood flesh [207], *auxin efflux carrier* family for fruit acidity [232], *endopolygalacturonase* for melting flesh and clingstone in peaches [233,234], yucca (YUC) protein for stony hard texture [202], and *R2R3 MYB* transcription factor for glabrous skin in peaches [235]. Other key genes and regulators of slow ripening [236], volatile compounds [237], and anthocyanin accumulation in the skin [210] have also been identified in peaches.

In apricots, the genes expressed during the ripening process related to aroma [14,108,238], taste [14,108], ethylene [35,42], carotenoids [42,239], phenylpropanoids [240], flavonoids [241], and anthocyanins [14] are being monitored. The monitoring analysis of apricot firmness loss is mainly performed post-harvest [120,242,243]. The ripening process has been compared between different species including apricots and Japanese apricots [244], as well as apricots and peaches [103], by using the peach microarray μPEACH v1.0 [103]. The validation of these candidate genes in different progenies for which expression is linked to a phenotype of interest may be used as molecular markers applied to MAS in apricot breeding programs. In the case of epigenetic regulation, the development of epigenetic marks linked to the associated traits should be of great interest.

## 8. Conclusions: Challenges and New Opportunities

Classical plant breeding is a long and tedious process involving the generation of large populations through controlled crosses of previously selected progenitors and the final selection of top individuals—the future new varieties. In the case of *Prunus* species, this process can take around 15 years. In these programs, enhanced fruit quality is one of the main breeding goals due the increasing interest in functional foods, new varieties, and, in particular, foods rich in antioxidants like anthocyanins and carotenoids, with bigger sizes, earlier or later ripening stages, better flavor and texture, attractive colors, and other traits that positively influence a consumer’s buying decisions. The management, phenotyping, and selection process of progenitors and seedlings are the main factors limiting the generation of new cultivars. In addition, the introgression of a novel trait in commercial crops requires several generations of crosses and selection, and it is particularly time-consuming when the crop of interest has a generation time of some years from seed to seed. In order to improve the efficiency and viability of fruit quality breeding programs in relation to parent and seedling selection, the implementation of molecular tools is an essential requirement, including the development of new MAS strategies based on the molecular knowledge of the determination of the phenotypic traits. Molecular efforts should be focused on the analysis of the molecular basis of the target traits and the implementation of molecular tools to be incorporated together with the classical activity of the visual selection of good descendants. The identification and mapping of candidate genes and alleles responsible for each phenotype, as well as molecular markers associated with them, are necessary to facilitate the selection of progeny plants with interest in a breeding program. Additionally, molecular markers allow for the easy identification of homozygous and heterozygous progeny without the need to verify the phenotype of fruits. On the other hand, metabolic engineering could be an alternative method to obtain improved quality traits in *Prunus* fruits. A common approach, when available, is to overexpress heterologous regulatory genes in the species of interest or endogenous regulators. This situation does mean that MAS will replace conventional breeding because this MAS should be used as a complementary technique. The practical learning that conventional breeding gives in the selection of new *Prunus* varieties is a complement of the molecular support. In this context, though there has been a significant shift of conventional breeders to molecular breeding, the target must be well-defined in a complete integrated work plan including DNA markers for selection, RNA markers for the monitoring of ripening, and the development of epigenetic marks linked to the traits of interest. This integrated strategy will facilitate the development and optimization of molecular markers to be applied to the field exploitation of the new varieties, offering and integrating complete technological techniques.

## Figures and Tables

**Table 1:** Main altered processes during the ripening of apricot fruit.

Changes	Character	Events
Biochemical	Color	Chlorophyll degradation
		Dismantling of photosynthetic apparatus
		Biosynthesis of anthocyanins Accumulation of carotenoids
	Texture	Solubilization of pectin and cellulose
		Starch hydrolysis
		Changes in protein content
		Hydration of cell walls
		Cell wall enzyme activity
	Flavor and aroma	Biosynthesis, accumulation and degradation of organic acids
		Biosynthesis, accumulation and degradation of sugars
		Acidity loss
		Production of volatile organic compounds (VOCs)
		Alcohol ester synthesis
Metabolic	Control of pathways	Increase in respiration ratio
		Ethylene biosynthesis
		Changes in the metabolism of starch and organic acids
		Altered regulation of existing metabolic pathways
Molecular	Gene expression	Ripening-specific messanger RNA (mRNA) synthesis
		Small and interference RNA appearance
		Disappearance of mRNAs DNA demethylation
	Protein expression	Synthesis of de novo ripening-specific proteins
		Disappearance of proteins
		Biosynthesis of allergenic compounds
		Increase pathogen susceptibility
